# Enantioselective transformation of fluoxetine in water and its ecotoxicological relevance

**DOI:** 10.1038/s41598-017-15585-1

**Published:** 2017-11-17

**Authors:** María Jesús Andrés-Costa, Kathryn Proctor, Marco T. Sabatini, Anthony P. Gee, Simon E. Lewis, Yolanda Pico, Barbara Kasprzyk-Hordern

**Affiliations:** 1 Environmental and Food Safety Research Group (SAMA-UV), Desertification Research Centre CIDE (CSIC-UV-GV), Faculty of Pharmacy, University of Valencia, Av. Vicent Andrés Estellés s/n, Burjassot, 46100 Valencia, Spain; 2 Department of Chemistry, University of Bath, Bath, BA2 7AY UK

## Abstract

European legislation focusing on water quality is expected to broaden to encompass several pharmaceuticals as priority hazardous substances. This manuscript aims to challenge current regulatory approaches that do not recognize stereochemistry of chiral pharmaceuticals by testing the hypothesis that environmental transformation and effects of chiral pharmaceuticals are stereoselective. Our experiments revealed that, while degradation of chiral fluoxetine (FL) in river water occurs via non-enantioselective photochemical and mildly-enantioselective microbial processes favoring the (*R*)-enantiomer, a pronounced enantioselectivity favoring (*S*)-FL (leading to the formation of (*S*)-NFL (norfluoxetine)) is observed during activated sludge treatment. Toxicity tests proved strong enantiomer-specific toxicity in the case of *Tetrahymena thermophila*, protozoa that are utilized during activated sludge treatment ((*R*)-FL is 30× more toxic than (*S*)-FL; (*S*)-NFL is 10× more toxic than (*S*)-FL). This is of paramount importance as preferential degradation of (*S*)-FL in activated sludge microcosms leads to the enrichment of FL with 30× more toxic (*R*)-FL and formation of 10× more toxic (S)-NFL. It is commonly assumed that a decreased concentration of FL leads to decreased biological impact. Our study proves that despite the overall decrease in FL concentration, accumulation of toxic (*R*)-FL and formation of toxic (*S*)-NFL leads to much higher than presumed toxicological effects.

## Introduction

Pharmaceuticals are a group of pollutants with growing evidence regarding their environmental impacts. European legislation focusing on water quality is expected to broaden to encompass several pharmaceuticals as priority hazardous substances. This manuscript challenges current regulatory approaches that do not recognize stereochemistry of chiral pharmaceuticals. Fluoxetine (FL, known as Prozac) is used here as an example.

FL is a diphenhydramine derivative and selective serotonin reuptake inhibitor (SSRI). It is used to treat a variety of mental health problems such as depression, panic, anxiety, or obsessive-compulsive symptoms. There was a 165% increase in the prescribing of antidepressant drugs in England between 1998 and 2012 (an average of 7.2% per year)^1^. Indeed, FL is the fourth most prescribed antidepressant in England, and accounts for 11.3% of all antidepressant drug use^2^.

FL is extensively metabolized to norfluoxetine (NFL) and several other metabolites such as FL glucuronide, NFL glucuronide, *para*-trifluoromethylphenol and hippuric acid. The principal metabolite, NFL, is formed by *N*-demethylation of FL. The potency and selectivity of NFL’s SSRI activity is similar to that of the parent drug. The elimination of FL accounts for 80% excreted in the urine (as 11.6% FL, 7.4% FL glucuronide, 6.8% NFL, 8.2% NFL glucuronide, >20% hippuric acid, 46% other) and approximately 15% excreted in the feces^3^.

Recent research studies have shown that most pharmaceuticals, including FL and NFL, enter the aquatic environment via (un)treated communal wastewater. Both FL and NFL have been detected in wastewater and receiving waters at levels ranging from ng L^−1^ to μg L^−1^ ^4–14^. Furthermore, they were found in the tissue of fish collected near municipal wastewater discharges. Both FL and NFL remain biochemically active in the environment and can have marked effects on the morphology, physiology, and behavior of different species^6,10,15–18^.

Despite some limited research on fate and effects of FL, there has been very little attention paid to the stereochemistry of FL and its possible environmental impacts. FL has one chiral carbon in its structure and as a result it exists in two enantiomeric forms as (*S*)-FL and (*R*)-FL. Similarly, NFL exists in two enantiomeric forms as (*S*)-NFL and (*R*)-NFL. Enantiomers of the same drug have identical physicochemical properties but may differ in their biological properties. Thus, chiral drugs can undergo stereoselective mechanisms controlling their fate such as distribution, metabolism and excretion, as these processes (due to stereoselective interactions of enantiomers with biological systems) usually favor one enantiomer over the other. This leads to process-dependent changes in the enantiomeric composition of chiral compounds^9^. Metabolism of FL was found to be enantioselective in humans, with the (*R*)-enantiomer being metabolized faster than (*S*)-enantiomer^19^. Additionally, due to different pharmacological activity, enantiomers of chiral drugs can differ in their biological actions, potency and toxicity^20^. Enantiomers of FL have similar potency as inhibitors of the serotonin reuptake pump in humans whereas enantiomers of NFL act differently, with (*S*)-NFL showing higher inhibition capacity^21^.

Ecotoxicity of FL (and other pharmaceuticals) is currently assessed for the racemate, as FL is marketed as a racemic mixture of two enantiomers. Environmental risk assessment (ERA) approaches need to be re-evaluated as they are based on a simplistic assumption that FL present in the environment is racemic^9^. Indeed, limited research indicates that FL and NFL are present in the aqueous environment as non-racemic mixtures, i.e. enriched with one enantiomer^5,22–26^. Furthermore, FL was found to undergo enantioselective transformation during wastewater treatment^5,27^. In a recent study, Barclay *et al*. (2011) found a slight enrichment of FL and NFL with (*S*)-enantiomer in both raw and treated wastewater^22^. In contrast, MacLeod *et al*. (2007) reported that in their monitoring study FL was enriched with (*R*)-enantiomer in wastewater indicating faster degradation of (*S*)-enantiomer during wastewater treatment^5,23^. Ribeiro *et al*.^28^ did not observe enantioselectivity in fluoxetine’s biodegradation in activated sludge. However, the same group observed enantioselective degradation favouring (*R*)-FL by Labrys portucalensis strain F11^29^.

FL is often used as a model compound for assessing SSRI impact on aquatic organisms such as zebrafish, Japanese medaka, goldfish, gulf toadfish, rainbow trout, fathead minnows and polychaete worms (*Capitella teleta*)^30–36^. FL was reported as toxic at low concentrations to several aquatic species^30,37–39^ but enantiomer-dependent toxicity was not considered. In fact, FL was proposed as one of 10 pharmaceuticals potentially dangerous for the environment^5^. Enantioselective toxicity of FL was demonstrated for *Primephales promelas* and *Tetrahymena thermophila*, where (*S*)-FL was found to be more toxic than its respective enantiomer^34,38^. On the other hand (*R*)-FL was considered more harmful to *Pseudokirchneriella subcapitata* ^38^. To the authors’ knowledge, there are no published reports on NFL toxicity to aquatic organisms at enantiomeric level. NFL was reported to be more active in humans than the parent compound^40^. Fuller *et al*.^21^ determined that enantiomers of NFL have markedly different potencies as inhibitors of the uptake of serotonin with (*S*)-NFL being more potent than the (*R*)-enantiomer in rats. It is therefore expected that NFL’s ecotoxicity to aquatic organisms might also be enantiomer-dependent.

The above discussion clearly indicates that current ERA approaches that do not recognize stereochemistry of chiral pharmaceuticals are inaccurate and could lead to incorrect conclusions being drawn regarding the ecotoxicological effects of chiral drugs. The limited work in the area of stereochemistry-induced fate and effects of FL (and pharmaceuticals in general) is mainly due to lack of enantioselective analytical methods as well as availability of (affordable) enantiomerically pure analytical standards. Such analytical methods and affordable enantiomerically pure standards are essential to gather accurate data needed for comprehensive ERA of these compounds.

The overarching aim of this study was to verify, for the first time, enantiomer-dependent fate and ecotoxicological effects of FL and its main metabolite NFL in the aquatic environment. To achieve the aim, the key components of this study were:

i) To synthesize single enantiomers of FL and NFL.
ii) To develop an analytical method for the detection and quantification of enantiomers of FL and NFL utilizing chiral liquid chromatography coupled with tandem mass spectrometry.
iii) To undertake, for the first time, mechanistic study of the degradation of FL and NFL formation in controlled river water and activated sludge simulating microcosm experiments.
iv) To verify, for the first time, enantiomer-specific toxicity of FL and NFL in aquatic species.

## Results

### Synthesis of enantiomerically pure FL and NFL enantiomers

**Figure 1:**
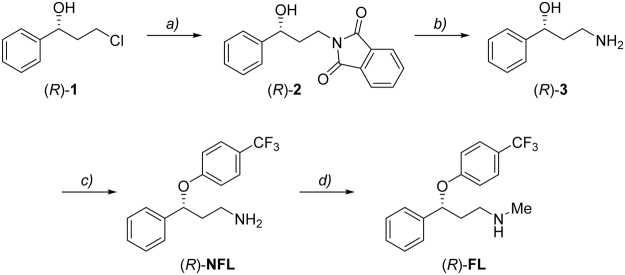
Synthesis of single enantiomers of NFL and FL. *Reagents and conditions*: (**a**) 1.2 equiv. potassium phthalimide, DMF, 90 °C, 2 h, 88%. (**b**) 3 equiv. NH_2_NH_2_^.^H_2_O, ethanol (EtOH), reflux, 2 h, 85%. (**c**) 1.5 equiv. sodium hydride, 1 equiv. *p*-fluorobenzotrifluoride, DMSO, 1 h, 90 °C, 71%. (**d**) 1.1 equiv. methyl chloroformate, 5 equiv potassium carbonate, CH_2_Cl_2_/H_2_O 1:2, 20 min, room temp., *then* 2 equiv. lithium aluminium hydride, THF, 2 h, reflux, 80%.

Lack of commercially available and affordable enantiomerically pure standards is the key factor hindering progress in the understanding of bio-physicochemical processes governing the fate and effects of chiral pharmaceuticals and the resulting environmental risks. To overcome this limitation and to enable toxicity studies on single-enantiomer drugs, we propose a conceptually straightforward approach. For any given drug of interest, our approach relies on taking a known chemical synthesis of the racemic form (for example, from the patent literature), and carrying out the same procedure, yet employing a single enantiomer of starting material. This removes the need to develop new synthetic procedures and allows for the rapid and cost-effective production of single enantiomer drug substance on a scale sufficient to enable toxicity studies. Careful choice must be given to the selection of the most appropriate literature synthesis, since the concept is not universally applicable – in some instances, a synthetic procedure might induce unwanted racemisation of one of the synthetic intermediates en route to the final drug substance. Nevertheless, in the case of FL and NFL, we have established a synthetic protocol that allows access to single enantiomers of NFL and FL, in just 3 or 4 synthetic steps, respectively. The single enantiomer starting material required (**1**) is commercially available in both the (*R*) and (*S*) forms (99% *e*.*e*.), and the cost is not prohibitive. Our protocol draws on multiple literature sources, as shown in Fig. 1. Thus, from single enantiomer starting material **1**, as per one of the original patents on fluoxetine^41^, reaction with potassium phthalimide affords single enantiomer **2**, which in turn undergoes hydrazinolysis to give single enantiomer **3**. Primary amine **3** can then be employed in a S_N_Ar reaction to give single enantiomer NFL. Various synthetic procedures are reported for the conversion of NFL into FL; we opted to employ carbamate formation followed by hydride reduction to convert a proportion of our synthesized NFL into FL^42^. By the above means we were able rapidly to access the required quantities of single enantiomer material to carry out the studies detailed below.

### Enantioseparation of FL and NFL enantiomers with chiral LC-MS/MS

**Figure 2:**
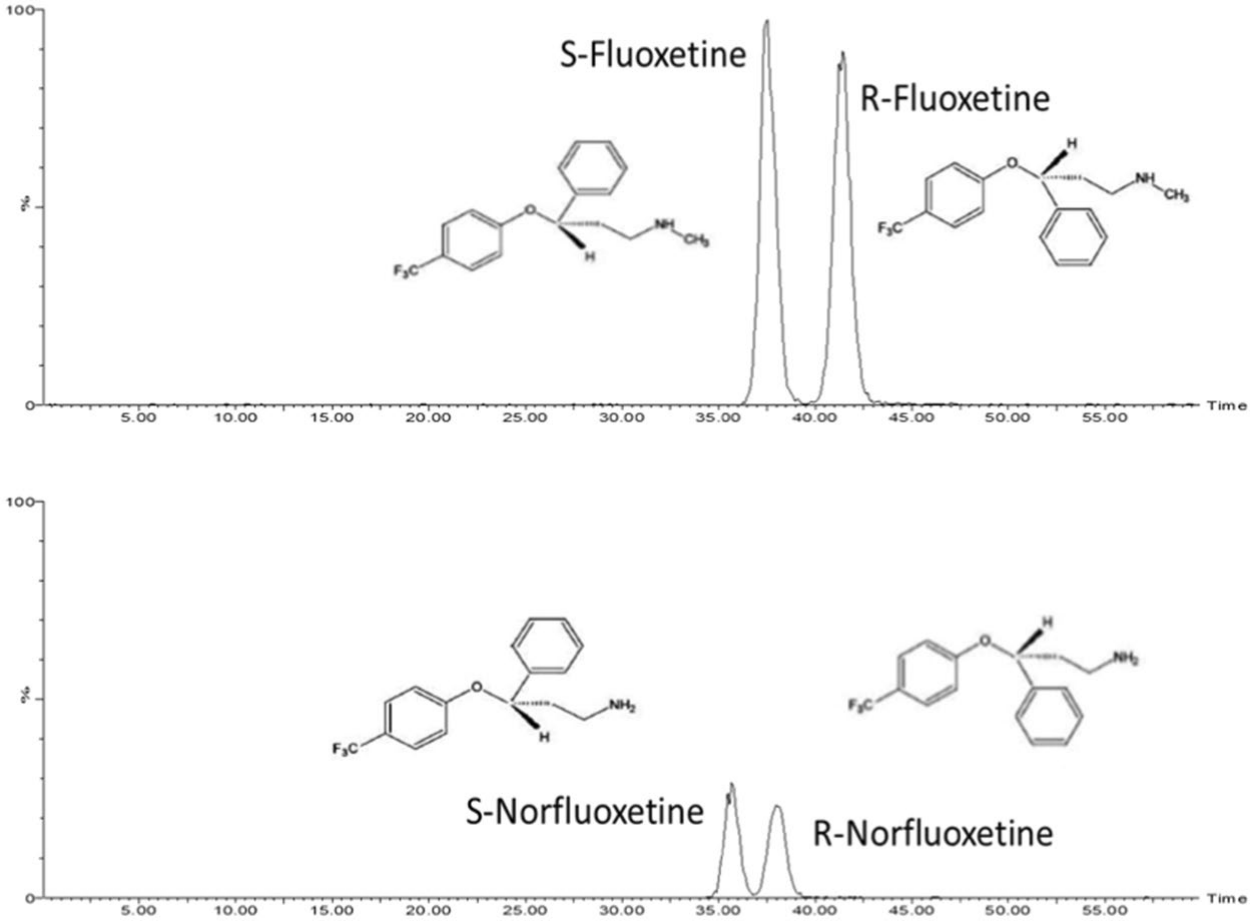
Chromatographic separation of enantiomers of FL and NFL.

Unavailability of analytical methods allowing for enantiomeric separations of trace concentrations of chiral drugs in complex environmental matrices is a limiting factor hindering progress in fundamental understanding of their fate and effects. We therefore developed a robust, sensitive and selective method utilizing chiral liquid chromatography coupled with tandem mass spectrometry for enantiomeric separation of FL and NFL. We used Astec Chirobiotic V (CBV) column with mobile phase (pH 6.5, 0.06 mL min^−1^, 25 °C) composed of 70% of ethanol (EtOH), 30% of ultra-pure water (HQ water), 4 mM of ammonium acetate (AAC) and 0.005% of formic acid (FA) under isocratic conditions to achieve baseline separation of enantiomeric pairs (Fig. 2, R_s_ (resolution of enantiomers) = 1.41 and 1.00 for FL and NFL‚ respectively). All conditions tested and results can be found in Table S1 in the supplementary information section. The method showed good linearity (R^2^ > 0.99) for all four enantiomers within the studied range (0.5–100 µg L^−1^). Method detection and quantification limits (MDLs and MQLs) for river water matrices ranged from 1.2 to 1.3 ng L^−1^ and from 4.6 to 5.1 ng L^−1^, respectively. In the case of activated sludge matrices, MDLs ranged from 0.4 to 0.8 ng L^−1^ and MQLs ranged from 1.7 to 3.1 ng L^−1^ (Table S2). The accuracy and precision were within ±20% (Table S3). Very good recoveries accounting for >67% were observed in the case of all four enantiomers in all studied matrices. Matrix effect (ME) accounted for <15.6%.

Synthesized enantiomerically pure FL and developed chiral LC-MS/MS method were used to verify transformation of FL in river and activated sludge simulating microcosms and their ecoxoticological impacts.

### Transformation of FL and NFL in river and activated sludge simulating microcosms

#### River water microcosms

**Figure 3:**
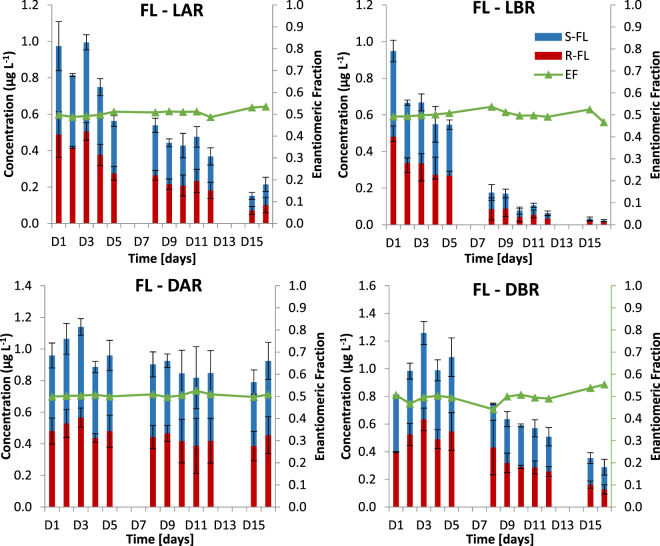
Degradation of FL in river water simulating microcosms under dark abiotic (DAR), dark biotic (DBR), light abiotic (LAR) and light biotic (LBR) conditions (concentrations are represented by bars, enantiomeric fractions are represented by symbols).

The river simulating microcosms revealed that degradation of FL takes place via both microbial and photochemical processes (Fig. 3 and Table S4). Photolysis is considered to be the most important phenomenon contributing to the degradation of FL, as 74.5% (*S*)-FL and 79.2% (*R*)-FL of FL were removed in light abiotic conditions (LAR). This process, as expected, was found not to be enantioselective. Microbial processes resulted in mild enantioselectivity towards (*R*)-enantiomer and led to the removal of 60.4% of (*S*)-FL and 67.9% of (*R*)-FL at dark biotic conditions (DBR). As expected, the light biotic reactor (LBR) utilizing both photochemical and microbial processes led to the highest removal of FL: 98.4% of (*S*)-FL and 96.7% of (*R*)-FL. Dark abiotic conditions (DAR) did not lead to any significant removal of FL. Traces of NFL were observed in both abiotic and biotic conditions (Figure S1). This indicates that degradation of FL leading to NFL formation takes place as a result of both photochemical and microbial processes.

#### Activated sludge microcosm

**Figure 4:**
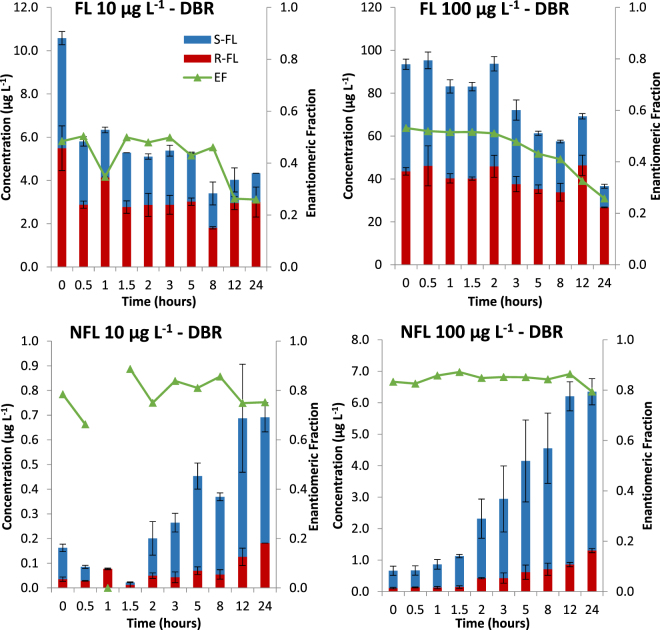
Degradation of FL and formation of NFL in activated sludge simulating microcosms under dark biotic (DBR) conditions (concentrations are represented by bars, enantiomeric fractions are represented by symbols).

Transformation of FL in activated sludge simulating microcosms was studied at two concentration levels: 10 and 100 µg L^−1^ of racemic FL (Fig. 4 and Table S5). In both cases a significant decrease in the concentration of (*S*)-FL and (*R*)-FL was observed. In the microcosm spiked with 10 µg L^−1^ rapid removal of FL occurred during the first 30 minutes (50% degradation). This process was not stereoselective (enantiomeric fraction (EF) 0.5) and did not lead to expected formation of NFL. It is therefore postulated that this high rapid removal of FL from the aqueous phase during the first 30 min of the experiment is due to its sorption to suspended particulate matter. Further removal of FL in 10 µg L^−1^ reactor was much slower and led to its stereoselective transformation favoring (*S*)-FL (EF < 0.3) and leading to the formation of NFL enriched with (*S*)-enantiomer (EF > 0.7). As the activated sludge simulating microcosms were undertaken in the dark, it is postulated that observed stereoselective transformation of FL and stereoselective formation of NFL is due to the prevalence of stereoselective microbial metabolic processes in studied bioreactors. Molar percentage yield of NFL formation denoted: 10.7% and 6.2% for (*S*)-FL and (*R*)-FL‚ respectively.

Similar observations were recorded in the microcosm spiked with 100 µg L^−1^ of FL. However, the effect of sorption was not observed. This is probably due to much higher initial FL load in 100 µg L^−1^ bioreactor not allowing for the change to be recorded. Stereoselective microbial processes resulted in 60% transformation of FL with twice as high preference towards (*S*)-FL (EF < 0.3, 80% removal of (*S*)-FL and only 38% removal of (*R*)-FL) and formation of NFL enriched with (*S*)-enantiomer (EF > 0.7). Molar percentage yield of NFL formation denoted: 11.7% and 7.4% for (*S*)-FL and (*R*)-FL. This is in agreement with results obtained for 10 µg L^−1^ bioreactor. Interestingly in both bioreactors, long lag phases (3 h and 2 h in the case of 10 µg L^−1^ and 100 µg L^−1^ FL bioreactor‚ respectively) were observed.

Kinetic studies (Table 1) confirmed low biodegradation of FL and the more recalcitrant nature of (*R*)-FL. k_biol_ and t_1/2biol_ of (*S*)-FL transformation were 0.04 Lg_SS_^−1^h^−1^ and 19 h respectively in both 10 and 100 µg L^−1^ bioreactors. k_biol_ and t_1/2biol_ of (*R*)-FL transformation were much lower and denoted 0.01 Lg_SS_^−1^h^−1^ and 68 h respectively in 100 µg L^−1^ bioreactors. Due to the lack of degradation of (*R*)-FL in 10 µg L^−1^ bioreactor, no kinetic studies could be undertaken.

**Table 1:** Degradation pseudo-first order rate constants (k_1_ and k_biol_) in single-compound activated sludge simulating microcosm.

		R^2^	SS [g L^−1^]	k_1_ [h^−1^]	t_1/2_ [h]	k_biol_ [Lg_SS_^−1^h^−1^]	t_1/2biol_ [h]
**10** **µg** **L** ^**−1**^							
(*S*)-FL	y = −0.0715x - 0.0584	0.8696	2.0	0.07	9.69	0.04	18.9
(*R*)-FL	No degradation	n/a	n/a	n/a	n/a	n/a	n/a
**100** **µg** **L** ^**−1**^							
(*S*)-FL	y = −0.0721x - 0.0667	0.8216	2.0	0.07	9.61	0.04	19.4
(*R*)-FL	y = −0.0206x - 0.0274	0.8202	2.0	0.02	33.6	0.01	68.0

n/a - not calculated due to no degradation of (*R*)-(−)-enantiomer.

The results above indicate that longer sludge retention times might be needed during wastewater treatment in order to facilitate degradation of FL. However, it should be noted that these processes are likely to be stereoselective and could potentially lead to enrichment of FL with the more potent enantiomer, as well as formation of biologically active metabolites; this is despite the nominal decrease in concentration levels of FL.

### Ecotoxicity of FL and NFL

Enantiomer-dependent toxicity of FL and NFL was evaluated for two aquatic organisms: *Daphnia* *magna* and *T* *etrahymena* *thermophila*. EC50_48h_ for FL enantiomers towards *D*. *magna* was 3.6 mg L^−1^ and 4.1 mg L^−1^ for (*S*)-FL and (*R*)-FL respectively (Table 2). EC50_48h_ for NFL towards *D*. *magna* denoted 2.8 mg L^−1^ and 2.9 mg L^−1^ for (*S*)-NFL and (*R*)-NFL (raw data are shown in Table S6). The results indicate a noticeable difference between the toxicity of FL and NFL, NFL being more toxic than FL, but no significant enantioselectivity was observed for studied enantiomeric pairs. In contrast, EC50_24h_ for FL enantiomers towards *T*. *thermophila* was strongly enantiomer dependent and denoted 35.3 mg L^−1^ and 1.3 mg L^−1^ for (*S*)-FL and (*R*)-FL‚ respectively. These results contradict those published by De Andrés *et al*.^38^ as within that study it was observed that the (*S*)-enantiomer was more toxic with an EC50_24h_ of  3.2 mg L^−1^ compared to the (*R*)-enantiomer with an EC50_24h_  30.5 mg L^−1^. To confirm the validity of measurement undertaken in this work, the stock solutions and the test cells were analyzed with the chiral-LC-MS/MS method. The results showed that the changes in the concentrations of the toxicants were minimal during the test, however they support the use of the correct enantiomer in this test. This was further confirmed by the use of enantiomerically pure analytical standards to confirm the retention time of each enantiomer (Figure S2).

**Table 2:** Ecotoxicity of FL and its metabolite NFL (n/a – not analysed).

Organism	Test	Toxicity endpoints	Effect [mg L^−1^]				Ref
			FL		NFL		
			(*S*)*-*	(*R*)*-*	(*S*)*-*	(*R*)*-*	
*P*. *promelas*	LOEC_7d_	-survival	0.10	0.17	n/a	n/a	34
		-growth	0.05	0.17	n/a	n/a	
		-feeding	0.05	0.17	n/a	n/a	
	LC50_48h_	-survival	0.22	0.21	n/a	n/a	34
*D*. *magna*	LOEC_21d_	-immobilization	0.44	0.43	n/a	n/a	34
		-reproduction	0.44	0.43	n/a	n/a	
		-grazing	0.20	none	n/a	n/a	
	LC50_48h_	-immobilization	6.9	8.1	n/a	n/a	38
	EC50_48h_	-immobilization	3.6	4.1	2.8	2.9	(this study)
*T*. *thermophila*	EC50_24h_	-growth	3.2	30.5	n/a	n/a	38
	EC50_24h_	-growth	35.2	1.3	3.8	5.8	(this study)

**Figure 5:**
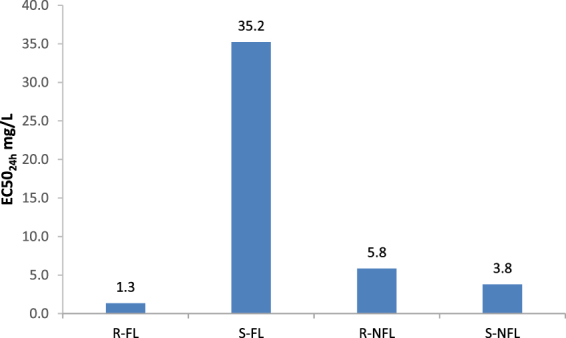
EC50_24h_ for the *T*. *thermophila* test. See Tables S7–14 for CV% of individual tests.

Similarly to FL, EC50_24h_ for NFL enantiomers towards *T*. *thermophila* was strongly enantiomer dependent. The EC50_24h_ for NFL denoted 3.8 mg L^−1^ and 5.8 mg L^−1^ for (*S*)-NFL and (*R*)-NFL‚ respectively. Unlike FL, the (*S*)-NFL is more toxic than (*R*)-NFL. This is of paramount importance as preferential degradation of less toxic (*S*)-FL in activated sludge microcosms leads to the formation (and accumulation) of more toxic (*S*)-NFL. This is also an important consideration as *T*. *thermophila* is part of the microbial community of activated sludge (EC50_24h_ data for *T*. *thermophila* is shown in Fig. 5 and Tables S7–S14).

## Discussion

This report is, to the authors’ knowledge, the first to study transformation of FL in environment simulating microcosms combined with ecotoxicological effects. The research reported in this manuscript tested and validated the hypothesis that degradation of FL, and formation of its main metabolite NFL, are enantioselective and biological in nature, and that their toxicity is enantiomer-dependent.

The river simulating microcosms revealed that degradation of FL takes place via both microbial and photochemical processes. Non-stereoselective photolysis was observed to be the most important phenomenon contributing to the degradation of FL. Microbial processes resulted in only mild enantioselectivity towards the (*R*)-enantiomer. However, a pronounced stereoselectivity was observed during activated sludge simulating microcosms. Microbial metabolic processes of FL during activated treatment process favored the (*S*)-enantiomer, which led to the enrichment of FL with the (*R*)-enantiomer. This is in contrast to metabolic processes in humans, which favor the (*R*)-enantiomer and lead to enrichment of FL in urine with the (*S*)-enantiomer^19^. The outcomes of human metabolism studies as well as full scale and microcosm wastewater treatment measurements indicate that enantiomeric signature of FL can change subject to composition and structure of microbial communities present in wastewater. Indeed in our full scale untreated wastewater study, FL was enriched with the (*S*)-enantiomer (EF 0.7)^43^. This confirms, yet again, complexity of environmental processes and reinforces the need for further comprehensive studies focusing on transformation of chiral pollutants in the environment.

Toxicity tests showed that while there is no significant enantioselectivity in the toxic response from *D*. *magna* to both FL and NFL, a strong enantiomer-dependent toxicity is observed in the case of *T*. *thermophila* ((*R*)-FL 30× higher than (*S*)-FL and (*S*)-NFL 10× higher than (*S*)-FL).

The above results indicate that traditional toxicological studies that do not recognize the importance of stereochemistry might not reveal the true toxicological impact resulting from stereochemistry of chiral drugs. Our research indicates that (*S*)-FL is preferentially degraded in activated sludge microcosms. This is expected, as (*S*)-FL is the least toxic to protozoa (organisms that are known to be key contributors to activated sludge treatment process) out of all four FL/NFL enantiomers studied. Unfortunately, this also indicates that FL, due to preferential metabolic degradation of (*S*)-FL, gets enriched with more toxic (*R*)-FL and leads to the formation of more toxic (*S*)-NFL. This accumulation of toxic (*R*)-FL and (*S*)-NFL will have detrimental effects on the performance of activated sludge treatment processes.

This study revealed that there are several, unaccounted for, underlying issues in both exposure and hazard assessment within ERA of chiral pharmaceuticals. One can assume that, if the stereochemistry of FL is not considered, decreased concentration of FL as a result of activated sludge treatment leads to decreased biological impact. Such an approach (as currently applied in ERA) can lead to false conclusions impacting environmental health. Our study proves that despite the overall decrease in FL concentration, accumulation of toxic (*R*)-FL and formation of toxic (*S*)-NFL in activated sludge will likely lead to higher toxicological effects, as observed in the case of protozoa. The European Medicines Agency guideline on the ERA of Medicinal Products for Human Use^44^ and the EU Directive for ERA for Veterinary Medicinal Products^45^ recommend the estimation of exposure and the prediction of risk calculation for the whole parent compounds only i.e. as a racemate or a mixture of stereoisomers if distributed as such. Therefore, current ERA leads to under or overestimation of toxicity of chiral pharmaceuticals and to incorrect ERA as chiral pharmaceuticals are present in the environment in their non-racemic forms and they show enantiomer-specific biological effects. We therefore recommend the adoption of a new strategy within ERA acknowledging stereochemistry of studied targets.

## Methods

### Chemicals and materials

HPLC-grade methanol (MeOH), EtOH, AAC, (99%), FA (98%) were purchased from Sigma Aldrich (Cambridge, UK). HQ water was supplied by a Milli-Q system (PURELAB, Elga, UK).

The reference standards, *rac*-FL and *rac*-NFL and the internal standard (IS) FL-d_5_ were purchased from LGC Standards (Teddington, UK). All standards and ISs were of the highest purity available (>97%). Structures, molecular formulae and molecular weights of target enantiomers are summarized in Table S15.

Stock solutions of the individual compounds were purchased in MeOH at a concentration of 1 mg mL^−1^ or 0.1 mg mL^−1^ and stored in the dark at −16 °C. Working solutions were prepared by diluting stock solutions in mobile phase or MeOH on a daily basis and stored at 4 °C.

All glassware was deactivated with dimethyldichlorosilane (5% DMDCS in toluene, Sigma-Aldrich) to minimize sample loss through adsorption of basic analytes onto –OH sites present on the glass surface^46^. Oasis HLB (60 mg, 3 mL, Waters, UK) were used for solid phase extraction (SPE). HQ water, river water (collected in South-West England) and activated sludge (collected from a local wastewater treatment plant) were used for method development and validation.

### Synthesis of FL and NFL enantiomers

This procedure employs 3-chloro-1-phenyl-1-propanol as starting material and source of chirality. This was purchased from Sigma-Aldrich chemical company: (*R*)-enantiomer product #338419; (*S*)-enantiomer product #324612. Both were certified as having 99% enantiomeric excess. Experimental procedures are described for the (*R*)-enantiomer; identical procedures were carried out with the (*S*)-enantiomer of starting material to synthesize (*S*)-NFL and (*S*)-FL.

#### Step 1: (R)-3-phthalimido-1-phenylpropanol (2)

At room temperature, to a stirring suspension of potassium phthalimide (3.93 g, 21.25 mmol) in dry dimethylformamide (DMF) (115 mL) was added (*R*)-3-chloro-1-phenyl-1-propanol **1** (3.00 g, 17.65 mmol) in dry DMF (5 mL). The reaction mixture was heated to 90 °C and left to stir for 2 hours, until completion was observed by thin layer chromatography (TLC.) To the cooled reaction mixture was added H_2_O (300 mL), and extracted with diethyl ether (2 × 300 mL). The combined organic extracts were washed with a saturated solution of LiCl (300 mL), brine (300 mL), dried over MgSO_4_ and filtered. The filtrate was concentrated *in vacuo* to give (*R*)-3-phthalimido-1-phenylpropanol **2** as a white powder (4.30 g, 88%); mp 78–79 °C; R_*f*_ 0.36 (3:1 Petrol/ethyl acetate). δ_H_ (250 MHz, CDCl_3_) 7.86–7.83 (2 H, m, ArH), 7.74–7.71 (2 H, m, ArH), 7.36–7.21 (5 H, m, ArH), 4.69 (1 H, t, *J* 6.5 Hz, CHOH), 3.91 (2 H, t, *J* 6.5 Hz, CH_2_N), 2.13–2.05 (2 H, m, CH_2_CHOH); δ_C_ (300 MHz, CDCl_3_) 168.8, 143.5, 134.0, 131.9, 128.4, 127.4, 125.6, 123.3, 71.2, 37.6, 34.8 (Figure S3).

#### Step 2: (R)-3-amino-1-phenyl-1-propanol (3)

At room temperature, to a stirred solution of (*R*)-3-phthalimido-1-phenylpropanol **2** (4.10 g, 14.5 mmol) in EtOH (90 mL) was added hydrazine hydrate (2.09 mL, 43.5 mmol). The reaction mixture was stirred for 1 hour and then heated to reflux for 2 hours. The reaction mixture became thick and cloudy upon heating, and when cooled, precipitate was filtered off. Recovered filtrate was concentrated under reduced pressure, diluted with dichloromethane (DCM) (10 mL) and filtered, washed with DCM (2 × 5 mL). The recovered filtrate was concentrated *in vacuo* to give the title compound (*R*)-3-amino-1-phenyl-1-propanol **3** as a brown oil (1.85 g, 85%); R_*f*_ 0.09 (100:10:1 DCM/MeOH/ Et_3_N). δ_H_ (300 MHz, dimethylsulfoxide (DMSO-*d*_6_)) 7.34–7.27 (4 H, m, ArH), 7.24–7.17 (1 H, m, ArH), 4.66 (1 H, dd, *J* 7.0, 6.0 Hz Hz CHOH), 2.71–2.60 (2 H, m, CH_2_N), 1.69–1.62 (2 H, m, CH_2_CHOH); δ_C_ (75 MHz, DMSO-*d*_6_) 146.6, 128.0, 126.6, 125.7, 71.3, 42.2, 38.9 (Figure S4).

#### Step 3: (R)-3-Phenyl-3-[4-(trifluoromethyl)phenoxy]-1-propanamine•HCl [(R)-NFL hydrochloride salt]

At 0 °C, to a stirred suspension of sodium hydride (60% in oil, 0.73 g, 18.34 mmol) in DMSO (3.0 mL) was added (*R*)-3-amino-1-phenyl-1-propanol **3** (1.85 g, 12.23 mmol) in DMSO (1.0 mL). The reaction mixture was stirred at 55 °C for 30 min and 4-fluorobenzotrifluoride (3.01 g, 18.34 mmol) in 1.85 mL DMSO was added dropwise. The resulting mixture was heated for 1 hour at 90 °C, until completion was observed by TLC. The mixture was cooled to 0 °C, and diluted with aqueous 1 N NaOH (20 mL). Toluene was used to extract the product (3 × 20 mL), and combined organic extracts were dried over MgSO_4_ and filtered. The crude product was purified by column chromatography (100:0:1 to 100:6:1 DCM/MeOH/Et_3_N) to give (*R*)-3-phenyl-3-[4-(trifluoromethyl)phenoxy]-1-propanamine [(*R*)-NFL] as a brown oil (2.10 g, 58%). Product (1.0 g, 3.39 mmol) was dissolved in 4 M HCl in dioxane (10.0 mL, 40 mmol), and left to stir for 2 hours. Reaction mixture was concentrated *in vacuo*, and recrystallized (50 mL of 3:2 diethyl ether/hexane) to give (*R*)-3-Phenyl-3-[4-(trifluoromethyl)phenoxy]-1-propanamine hydrochloride [(*R*)-NFL•HCl] as a white solid (0.80 g, 71%); mp 128–129 °C; R_*f*_ 0.04; δ_H_ (250 MHz, CDCl_3_) 8.45 (3 H, br s, NH_3_) 7.39 (2 H, d, *J* 8.5 Hz, ArH), 7.27–7.32 (5 H, m, ArH), 6.91 (2 H, d, *J* 8.5 Hz, ArH), 5.42 (1 H, dd, *J* 7.5, 4.5 Hz, CHO), 3.18 (2 H, app t, *J* 5.5 Hz, CH_2_CH_2_NH), 2.47–2.27 (2 H, m, CH_2_N); δ_C_ (300 MHz, CDCl_3_): 159.5, 139.0, 129.1, 128.4, 126.7 (q, ^3^ *J*_CF_ 3.8 Hz), 125.7, 124.2 (q, ^1^ *J*_CF_ 270 Hz), 123.3 (q, ^2^ *J*_CF_ 32.6 Hz), 115.9, 77.4, 36.9, 36.0; ν_max_ (film) 3385 (N-H), 2891 (C-H), 2015, 1613 cm^−1^; [α]_D_ + 14.0° (*c* 1, CHCl_3_); for (*S*)-enantiomer: [α]_D_ –15.0° (*c* 1, CHCl_3_) (Figure S5).

#### Step 4: (*R*)-*N*-Methyl-3-(4-trifluoromethylphenoxy)-3-phenylpropylamine [(*R*)-FL]

At room temperature, to a stirred solution of 3-phenyl-3-[4-(trifluoromethyl)phenoxy]-1-propanamine (*R*)-NFL (1.0 g, 3.38 mmol) and methyl chloroformate (0.29 mL, 3.72 mmol) in DCM (15.0 mL) was added aqueous K_2_CO_3_ (2.33 g, 16.89 mmol in 30 mL H_2_O). The reaction mixture was vigorously stirred for 20 minutes, until completion was observed by TLC, dyed with ninhydrin. The organic phase was separated and the aqueous phase extracted with DCM (2 × 30 mL). The combined organic extracts were dried over MgSO_4_ and filtered. The filtrate was concentrated *in vacuo* to yield intermediate carbamate as a pale yellow oil. At 0 °C, to a stirring suspension of LiAlH_4_ (0.25 g, 6.59 mmol) in dry tetrahydrofuran (THF) (15.0 mL) was added dropwise a solution of the intermediate carbamate in dry THF (5.0 mL). The reaction mixture was gradually heated to reflux for 2 hours. To the cooled mixture were cautiously added 0.25 mL of water, followed by 0.25 mL of 2 N NaOH, and 0.75 mL of water, in that order. The solution was dried over MgSO_4_ and filtered. The filtrate was concentrated over reduced pressure, and the crude product was purified by column chromatography (100:0:1 to 100:1:1 EtOAc/MeOH/Et_3_N) to give (*R*)-*N*-Methyl-3-(4-trifluoromethylphenoxy)-3-phenylpropylamine [(*R*)-FL] as a pale yellow oil (0.84 g, 80%); R_*f*_ 0.09 (100:1:1 EtOAc/MeOH/Et_3_N); δ_H_ (300 MHz, CDCl_3_) 7.43 (2 H, d, *J* 8.5 Hz, ArH), 7.34–7.23 (5 H, m, ArH), 6.90 (2 H, d, *J* 8.5 Hz, ArH), 5.31 (1 H, dd, *J* 8.0, 4.5 Hz, CHO), 2.82–2.66 (2 H, m, CH_2_CH_2_NH), 2.44 (3 H, s, CH_3_), 2.27–2.16 (1 H, m, CHHN), 2.08–1.97 (1 H, m, CHHN), 1.69 (1 H, br. s, NH); δ_C_ (300 MHz, CDCl_3_) 160.5, 141.0, 128.8, 127.8, 126.7 (q, ^3^ *J*_CF_ 3.8 Hz), 125.7, 115.7, 78.5, 48.2, 38.6, 36.4 (signals for -CF_3_ and C-CF_3_ were not observed); ν_max_ (film) 3033 (ArC-H), 2937 (ArC-H), 2846 (C-H), 2796, 1614 cm^−1^; [α]_D_ + 3.0° (*c* 1, CHCl_3_); for (*S*)-enantiomer: [α]_D_ – 3.0° (*c* 1, CHCl_3_) (Figure S6).

### Microcosm bioreactors

#### River water simulating microcosms

**Figure 6:**
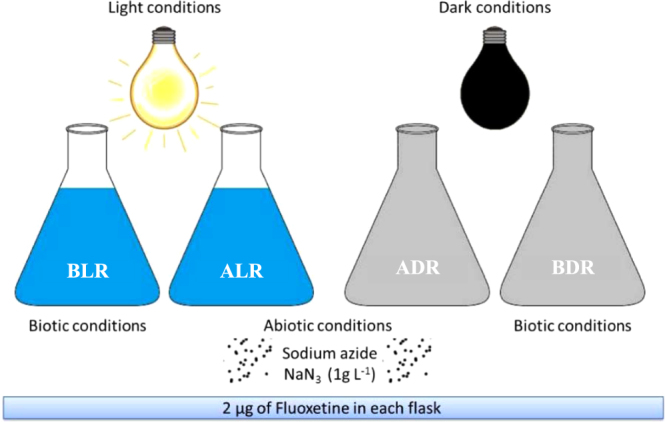
Scheme of river and activated sludge simulating microcosms.

River water microcosm experiments were conducted in light (L) and dark (D) conditions, and biotic (B) or abiotic (A) conditions with or without sodium azide (an inhibitor of microbial processes) respectively as shown in Fig. 6. Four microcosm bioreactors were investigated in duplicate thus eight autoclaved conical flasks were used as bioreactors in microcosm experiments. Each bioreactor was filled with 2 L of river water collected from a local river and spiked with racemic standard of *S*/*R* (±) FL to obtain a final concentration of 1 µg L^−1^. Abiotic bioreactors were spiked with sodium azide at a concentration of 1 g L^−1^ to inhibit biotic processes. Two replicates of biotic and abiotic bioreactors were exposed to light and another two replicates of each bioreactor were kept in the dark. Light conditions were simulated with an Osram 400 W HQI BT daylight lamp during 8 h each day. Average photon flux measured at the level of the bottle base was 395 µmol m^−2^ s^−1^. Dark conditions were simulated covering up the flask with foil. Magnetic stirrers were used to ensure good mixing. The experiment was carried out during 16 days. Samples (50 mL each) were collected every day, with the exception of weekends. IS was added to each sample (to obtain a final concentration of 100 ng mL^−1^). Samples were then frozen to prevent compound degradation until their analysis.

Dissolved oxygen (DO), pH and temperature (T) were analyzed during sampling period and total suspended solids (TSS), NO_2_^−^, NH_4_, and chemical oxygen demand (COD) were analyzed at the beginning of the experimental period (Table S16).

#### Activated sludge simulating microcosms

Activated sludge microcosm experiments were conducted in the dark and aerobic conditions. Three microcosm bioreactors were investigated in duplicate. They were filled with 2 L of fresh activated sludge collected from a local wastewater treatment plant. One bioreactor remained un-spiked and the other two bioreactors were spiked with a racemic standard of *S*/*R* (±) FL to obtain concentrations of either 10 or 100 µg L^−1^. Dark conditions were simulated by covering up the flask with foil. Aerobic conditions were obtained using air from BOC air cylinder and a thorough mixing was maintained using magnetic stirrers. The experiment was 24 hours long. Samples (100 mL each) were taken at the following time intervals: 0, 0.5, 1, 1.5, 2, 3, 5, 8, 12 and 24 h. After sample collection, IS was added to each sample to obtain a final concentration of 100 ng mL^−1^. Collected samples were subsequently frozen until their analysis to prevent degradation of compounds.

DO, pH, T and TOC, were analyzed throughout the experimental period and TSS, NO_3_^−^, NO_2_^−^, NH_4_ and COD were analyzed at the beginning of the experiment at t = 0 min (Table S17).

#### Kinetics – activated sludge simulating microcosms

The compounds studied are characterized as having low volatility and therefore volatilization was not considered as a potential removal pathway in studied microcosms. Photodegradation was also not considered (not relevant) under tested activated sludge conditions. Therefore the two important degradation mechanisms to consider were biodegradation and sorption to sludge. FL was reported to have high sorption affinity towards particulate matter^47^. However, in this study, sorption equilibrium was assumed and therefore sorption could be considered negligible. This is because sorption is assumed to be fast when compared to biological degradation^48^. Section “*Activated sludge microcosm*” confirms this hypothesis as FL in activated sludge microcosms remained constant in FL 100 µg L^−1^ microcosm during the first 2 h of the experiment. In the case of FL 10 µg L^−1^, a significant drop of FL concentration took place during the first 0.5 h (likely due to sorption) and then remained stable during the first 3 h of the experiment, showing a significant lag phase.

Several reports utilized pseudo-first-order kinetics for degradation of micropollutants in activated sludge reactors^48–50^. Indeed when applying pseudo-first order kinetics (OECD 303) in this work, ln(C_e_/C_i_) plotted as a function of time yielded a straight line (R^2^ > 0.9). Pseudo-first order biodegradation rate k_1_ [L g^−1^h^−1^] (normalised for concentration of suspended solids) was therefore calculated using the following formula (equation (1)):

1 $$In\,\frac{{C}_{e}}{{C}_{i}}=-{k}_{1}\ast t\ast SS$$

where: t = aeration time (24 h), C_e_ = concentration at time point t (µg L^−1^), C_i_ = initial concentration (µg L^−1^), SS = concentration of activated sludge solids (g L^−1^).

### Analysis

#### Solid phase extraction

Samples (50 mL of river water and 100 mL of activated sludge) were filtered using Whatman GF/F 0.7 µm glass fiber filter and passed through Oasis HLB cartridges (60 mg, 3 mL) pre-conditioned with 2 mL of MeOH and equilibrated with 2 mL of HQ water at a rate of 8 mL min^−1^. Samples were passed through the HLB cartridge at a rate of 8 mL min^−1^ and then dried under the vacuum for 30 min to dry out residual water. Analytes were eluted with 4 mL of MeOH at a rate of <1 mL min^−1^. Extracts were then evaporated to dryness with TurboVap evaporator (Caliper, UK, 40 °C, N_2_, <5 psi) and reconstituted in 0.5 mL of mobile phase. All samples were filtered through 0.2 µm PTFE filters (Whatman, Puradisc, 13 mm) and transferred to popylpropylene 0.3 mL capacity vials (Waters, UK).

SPE recoveries of FL and NFL in HQ water, river water and activated sludge were calculated as the ratio of the analyte peak area in the sample extract spiked with analytes before extraction (the peak area of analyte un-spiked sample extract was subtracted) to the analyte peak area in the non-extracted standard solution.

ME was calculated for each chiral drug as a percentage decrease or increase in signal intensity in a sample matrix versus HQ water using the following equation (2):

2 $$ME=\frac{\Delta matrix}{\Delta HQ\,water}$$

where Δmatrix is the standard calibration graph slope in different matrix (river water or active sludge) and ΔHQ water is the standard calibration graph slope in HQ water.

#### Chiral-LC-MS/MS method

Chromatographic analysis was performed using an Acquity UPLC system (Waters, Manchester, UK) consisting of Acquity UPLC binary solvent manager and Acquity UPLC sample manager. To achieve suitable separation of FL and NFL and their two stereoisomers, two chiral columns, namely Chiralpak CBH (10 cm × 2.0 mm, 5 μm particle size) and Astec CBV (25 cm × 2.1 mm, 5 μm particle size) were screened.

Several mobile phases were tested in order to obtain chiral separation of FL and NFL using LC and to maintain satisfactory electrospray ionization (ESI) performance in the positive ionization mode. MeOH, EtOH and HQ water were used at different concentrations as the mobile phase solvents. Among the mobile phase additives, different concentrations of AAC (1, 4 and 10 mM), MeOH (85, 80 and 70%) and EtOH (70 and 80%) were tested to maximize the R_s_ of FL and NFL.

The composition of the mobile phase was optimized to enhance the chromatographic efficiency and resolution between the enantiomers. R_s_ was calculated using the following equation (3):

3 $${R}_{S}=\frac{2(t{r}_{E2}-t{r}_{E1})}{{W}_{E2}+{W}_{E1}}$$

where tr_E1_ and tr_E2_ are the retention times of the first and the second eluted enantiomers, respectively, and W_E1_ and W_E2_ are the widths of these responses at the base peak.

The best enantiomeric separation of the studied drugs was achieved with a mobile phase (pH 6.5) composed of 70% of EtOH, 30% HQ water, 4 mM of AAC and 0.005% of FA using an Astec CBV column. The separation of enantiomers of chiral pharmaceuticals was undertaken under isocratic conditions, with an injection volume of 20 µL. The column was kept at 25 °C and the temperature in the sample manager was kept at 4 °C. The flow rate was 0.06 mL min^−1^, which gave an initial pressure of ~850 psi.

Identification and quantification of FL and NFL was undertaken with an Acquity Xevo TQD (Waters, Manchester, UK), a triple quadrupole MS equipped with an ESI source. The analyses were performed in positive mode with a capillary voltage of 3 kV, a source temperature of 150 °C and a desolvation temperature of 250 °C. A cone gas flow of 50 L h^−1^ and desolvation gas flow of 450 L h^−1^ were used. Nitrogen, used as a nebulising and desolvation gas, was provided by a high purity nitrogen generator (Peak Scientific Instruments Ltd., UK). Argon (99.99%) was used as a collision gas. The mass spectrometer was operated in multiple reaction monitoring (MRM) mode, measuring the fragmentation of the protonated pseudo-molecular ions of each compound. A dwell time of 20 ms per ion pair was optimized to maintain high sensitivity of the analysis. MassLynx v4.1 (Waters, UK) software was used to collect and analyze the obtained data.

Quantifier and qualifier transitions were optimized for each compound based on the most intense signal. Specific parameters such as collision energy (CE) and cone voltage (CV) were optimized for FL, NFL and FL-d5 separately in a continuous flow mode through direct injection of standard solution at a concentration of 50 µg L^−1^ into the stream of the mobile phase. FL presents a precursor ion [M + H]^+^ m/z of 310.3 and a product ion of m/z 44.2 (quantifier transition) with a CV of 34 and CE of 10 and m/z 148.2 (qualifier transition) with a CV of 25 and CE of 8. NFL presents a precursor ion [M + H]^+^ m/z 298.4 and a corresponding product ions at m/z 134.1 (quantifier transition) with a CV of 17 and CE of 7 and m/z 30 (qualifier transition) with a CV of 17 and CE of 7. FL-d5 presents a precursor ion [M − H]^+^ m/z 315.2 and a corresponding product ions at m/z 136.2 (quantifier transition) with a CV of 26 and CE of 71 and m/z 20.

### Method validation parameters

A 10-point multi-component IS calibration curve was applied for quantification of FL and NFL enantiomers. All instrumental and method validation parameters such as linearity and range, accuracy, precision, detection and quantification limits and calibration curve were determined for HQ water, river water and activated sludge spiked with known concentrations of chiral compounds.

Linearity and range of the analytical procedure were undertaken by serial dilution of stock solution (10 µg mL^−1^). Accuracy of the method was evaluated at three concentration levels (5, 25 and 250 µg L^−1^) as a percentage deviation from known added quantity of each enantiomer in the sample. Intra-day and inter-day precision was expressed by the relative standard deviation (RSD) of 3 replicate measurements at three different concentration levels (5, 25 and 250 µg L^−1^) on the same and three different days.

HQ water standard solutions were used for instrumental detection limit (IDL) and instrumental quantification limit (IQL). The IQL was estimated for the concentration of a compound that gave a signal-to-noise ratio of 10:1. The IDL corresponded to the concentration that gave a signal-to-noise of 3:1.

MDL and (MQL for river water and activated sludge were calculated using the following equations (4) and (5):

4 $$MDL=\frac{{ID}{{L}}_{S/N}\times 100}{{Re}c\times CF}$$

5 $$MQLcalc=\frac{{ID}{{L}}_{S/N}\times 100}{{Re}c\times CF}$$

where IDL_S/N_ is the instrumental detection limit (µg L^−1^), IQL_S/N_ is the instrumental quantification limit (µg L^−1^), Rec is the absolute recovery of the analyte (%) at 25 µg L^−1^, and CF is the concentration factor, which in this method denotes 200 for active sludge and 100 for river water.

The EF of studied chiral drugs was calculated using the following equation (6):

6 $$EF=\frac{(S)}{(S)+(R)}$$

Where (*S*) and (*R*) are concentrations of the (*S*)- and (*R*)- enantiomers, respectively.

### Toxicity tests

#### Daphnia magna acute 48 h immobilization assay

The *D*. *magna* bioassay was carried out using Daphtoxkit^TM^ (Crustacean Toxicity Screening Test for Freshwater; Microbiotests, Nazareth, Belgium) following the standard operational procedure in accordance to the ISO standard 6341:2012 and the OECD 202 guideline. Less than 24 h old daphnids were exposed to a series of concentrations of each enantiomer of FL and NFL. Six concentration levels (5 concentrations plus control, four replicate beakers for each concentrations, five individual for each beaker) were tested. The concentrations were from 0.5 to 50 mg L^−1^ for FL and NFL enantiomers based on preliminary range finding tests. Each experiment was repeated in triplicate. After 48 h incubation, daphnids were observed and the mobile daphnids in each container were reported. The EC50 were calculated using 48 h results.

#### Tetrahymena thermophila chronic 24 h population growth assay

*T*. *thermophila* bioassay was carried out using Protoxkit F (Microbiotests, Ghent University, Belgium). The tests were performed in accordance with the protocols provided by the manufacturer. Protoxkit F is a 24 h chronic population growth assay.

The tests were carried out in disposable spectrophotometric cells of 1 cm path-length, to enable the measurement of the optical density (OD) at 440 nm. These measurements were taken at T0h and T24h, as well as two hour increments after initial 24 h incubation to monitor the change in turbidity of the sample. The reconstituted food substrate supplied with each test provides an initial high turbidity at T0h, which, in the control cells, drastically decreases over the next 24 h due to the uninhibited growth of the ciliate population. This change in OD over the time period is used to quantify the degree of inhibition and subsequent calculation of the EC50. Each concentration was repeated in duplicate.

The initial protozoa inoculum was prepared by measuring an aliquot of the live suspension using photometry absorbance at 440 nm and diluted to achieve a theoretical OD value of 0.040. Each test cell is inoculated with 40 µL of this suspension, lending to an approximate population density of 100 protozoa per milliliter.

An initial study was carried out to find the approximate range of uninhibited growth and 100% inhibition for each enantiomer/compound across 7 orders of magnitude. The definitive toxicity test was carried out from the lowest concentration with a percentage population growth inhibition of 80–100% to the highest concentration with an inhibition between 0–20%. To ensure the test was valid the control must reach 60% OD decrease after 24 h. In some tests this may take 2–4 h longer than 24 h, this is batch dependent and indicates a slightly slower growth of the ciliates, however this is still considered valid. In this case all the tests carried out were with the same batch which took 28 h to fulfill the validation criteria. The EC50 values were calculated using 28 h results.

## Electronic supplementary material

Supplementary information
