# Supplementary material for: Enantioselective transformation of fluoxetine in water and its ecotoxicological relevance
Source: Sci Rep. 2017 Nov 17;7:15777. doi: 10.1038/s41598-017-15585-1 (PMC5693942; doi:10.1038/s41598-017-15585-1)
Supplement: Supplementary file 1 — Supplementary information [file 41598_2017_15585_MOESM1_ESM.pdf]

Enantioselective biodegradation of fluoxetine in water and its ecotoxicological relevance

María Jesús Andrés-Costa[1,2], Kathryn Proctor[2], Marco Sabatini[2], Anthony P. Gee[2], Simon E. Lewis[2], Yolanda Pico[1], Barbara Kasprzyk-Hordern[2*]

[1]*Environmental and Food Safety Research Group (SAMA-UV), Desertification Research Centre CIDE (CSIC-UV-GV), Faculty of Pharmacy, University of Valencia, Av. Vicent Andrés Estellés s/n, Burjassot, 46100 Valencia, Spain*

[2]*Department of Chemistry, University of Bath, Bath BA2 7AY, UK*

[*]Corresponding author: E-mail: b.kasprzyk-hordern@bath.ac.uk; Fax: +44(0) 1225 386231; Tel: +44 (0) 1225 385013

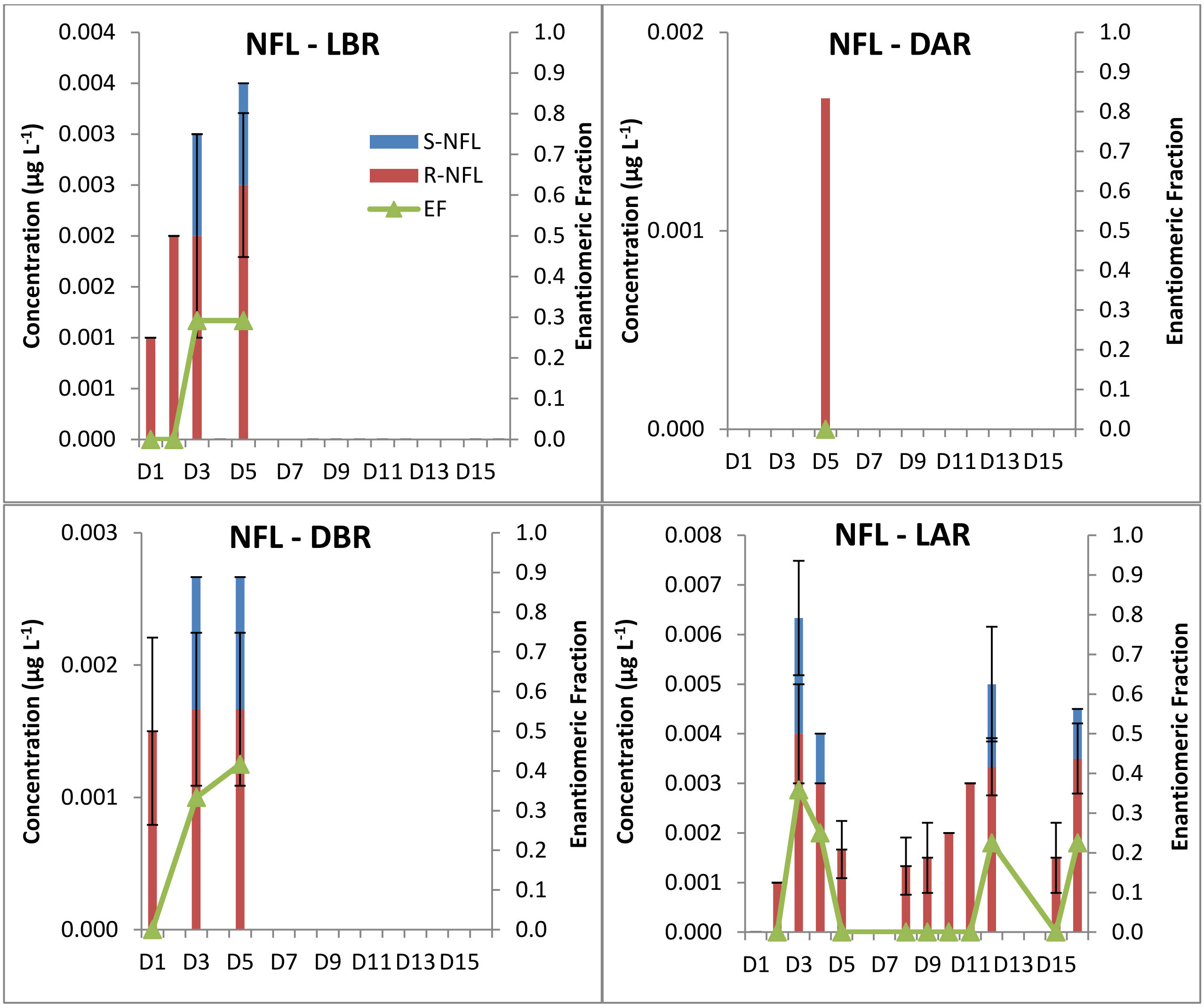

Figure S1. Formation of NFL during FL degradation in river simulating microcosms under dark abiotic (DAR), dark biotic (DBR), light abiotic (LAR) and light biotic (LBR) conditions (concentrations are represented by bars, enantiomeric fractions are represented by symbols).

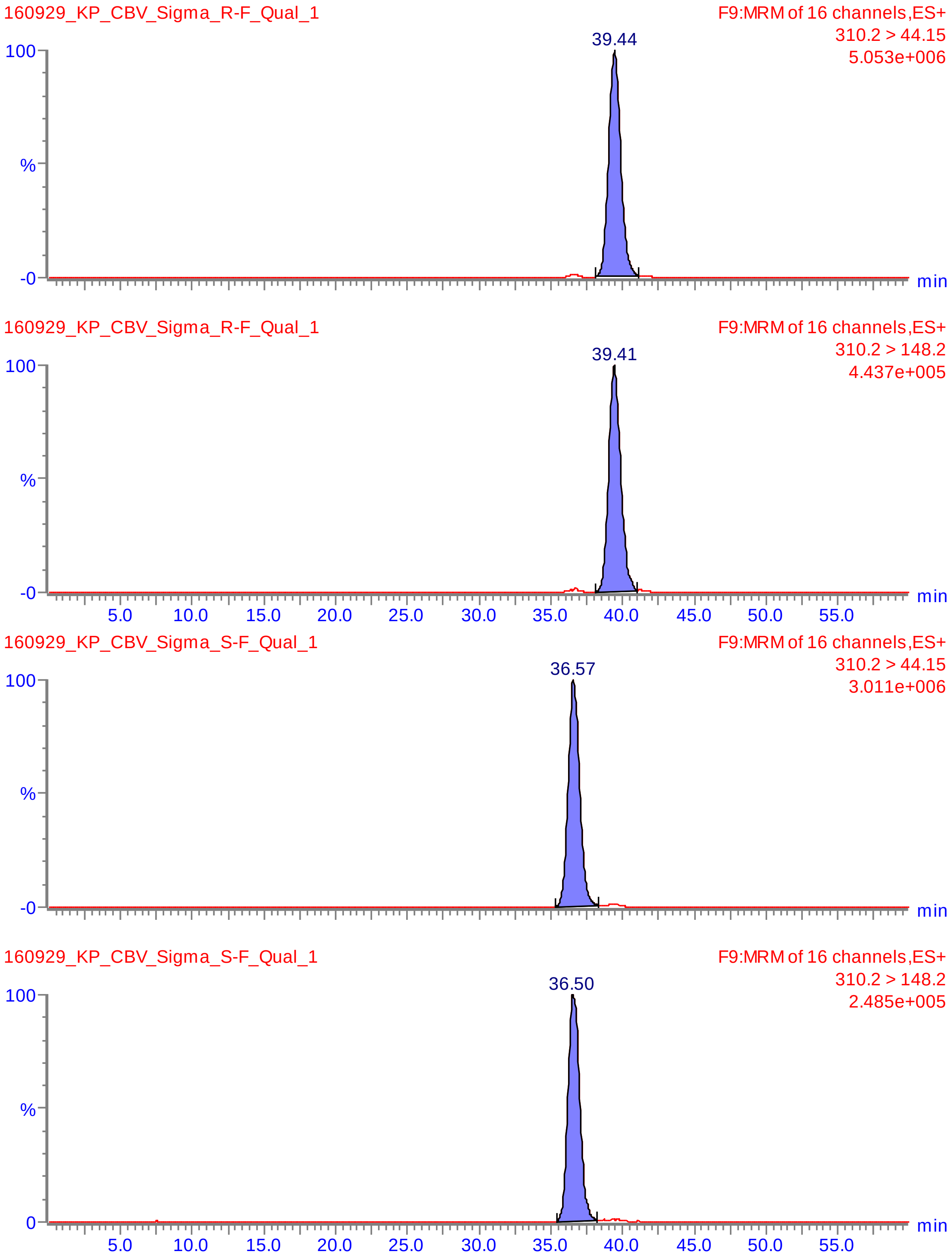

Figure S2. Qualitative confirmation of the retention time for Sigma enantiomerically pure FL standards. In order from top to bottom *R*-FL MRM1 RT = 39.44; *R*-FL MRM2 RT = 39.41; *S*-FL MRM1 RT = 36.57; *S*-FL MRM2 =36.50.

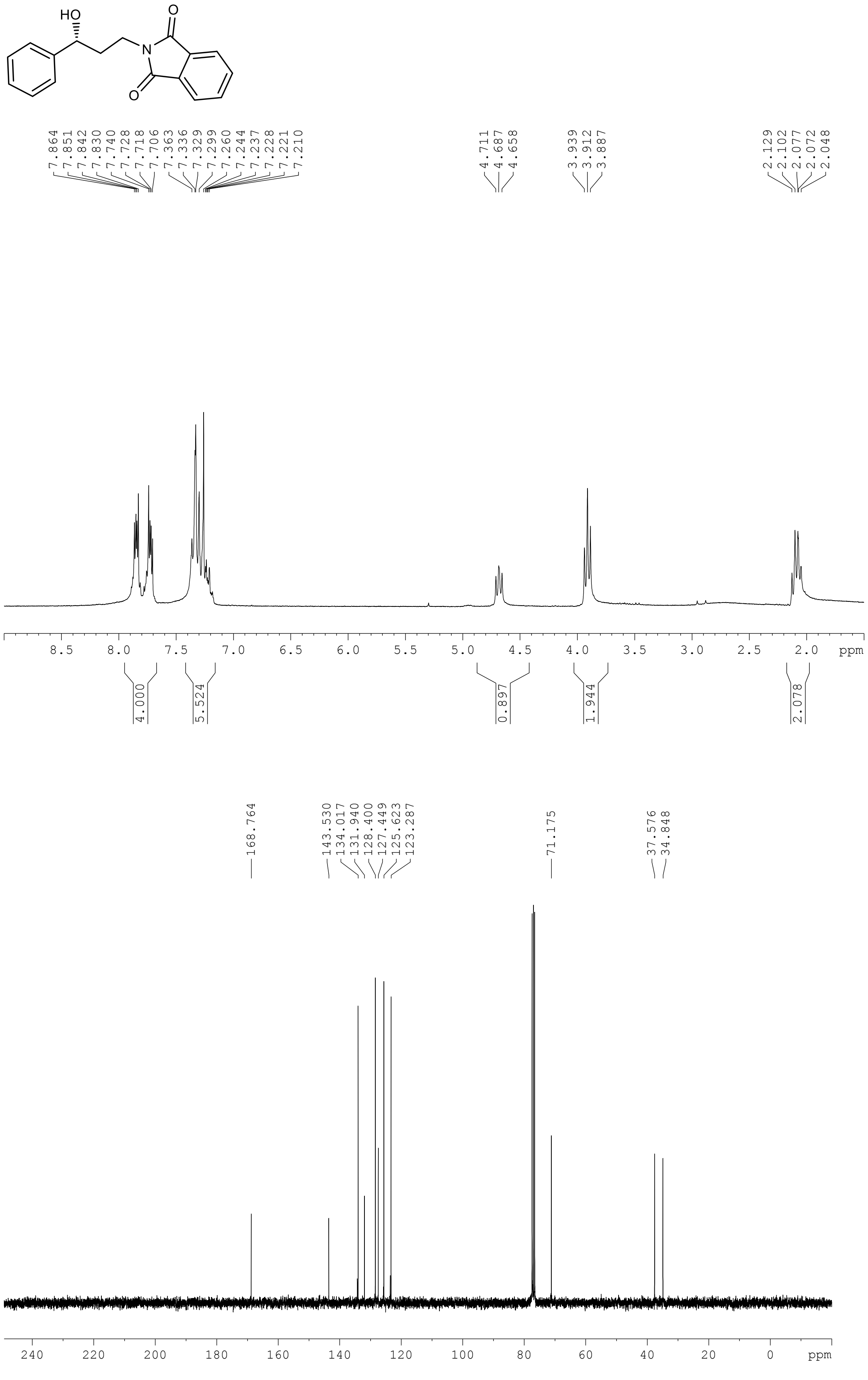

Figure S3. ^{1}H (upper) and ^{13}C NMR (down) average spectra of (*R*)-3-phthalimido-1-phenylpropanol

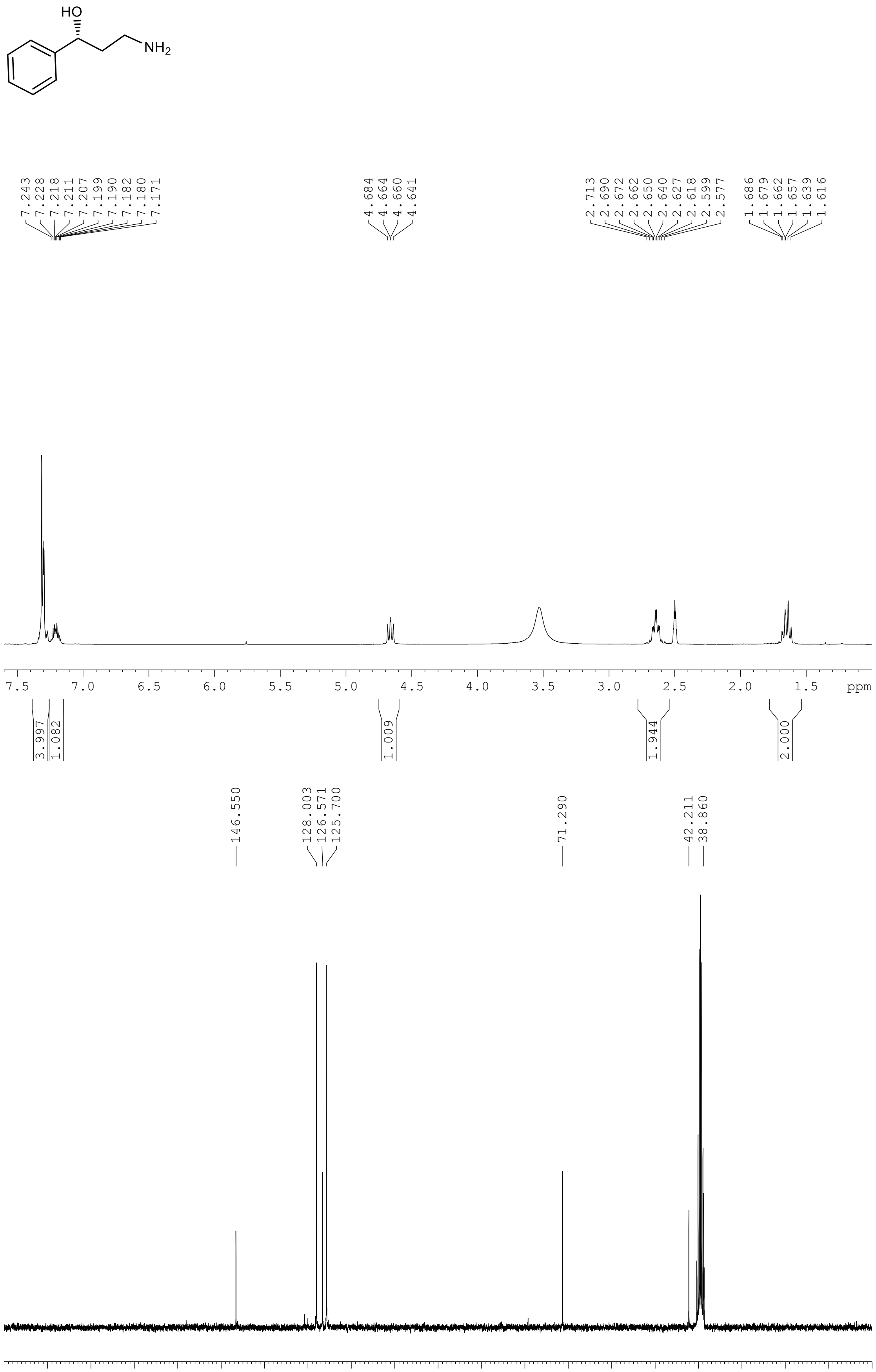

Figure S4. ^{1}H (upper) and ^{13}C NMR (down) average spectra of (*R*)-3-amino-1-phenyl-1-propanol

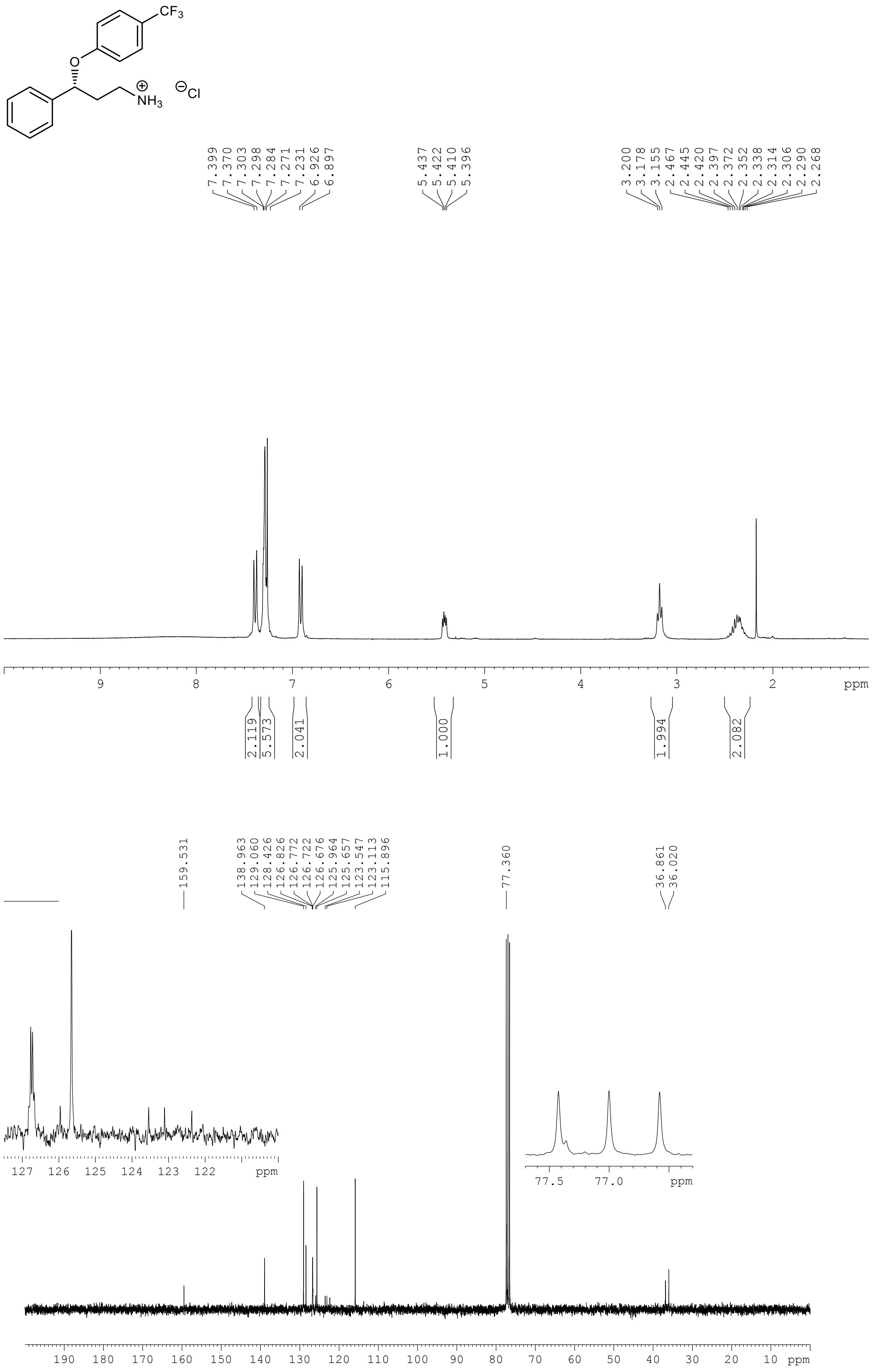

Figure S5. ^{1}H (upper) and ^{13}C NMR (down) average spectra of (*R*)-3-Phenyl-3-[4-(trifluoromethyl)phenoxy]-1-propanamine•HCl (*R*-NFL)

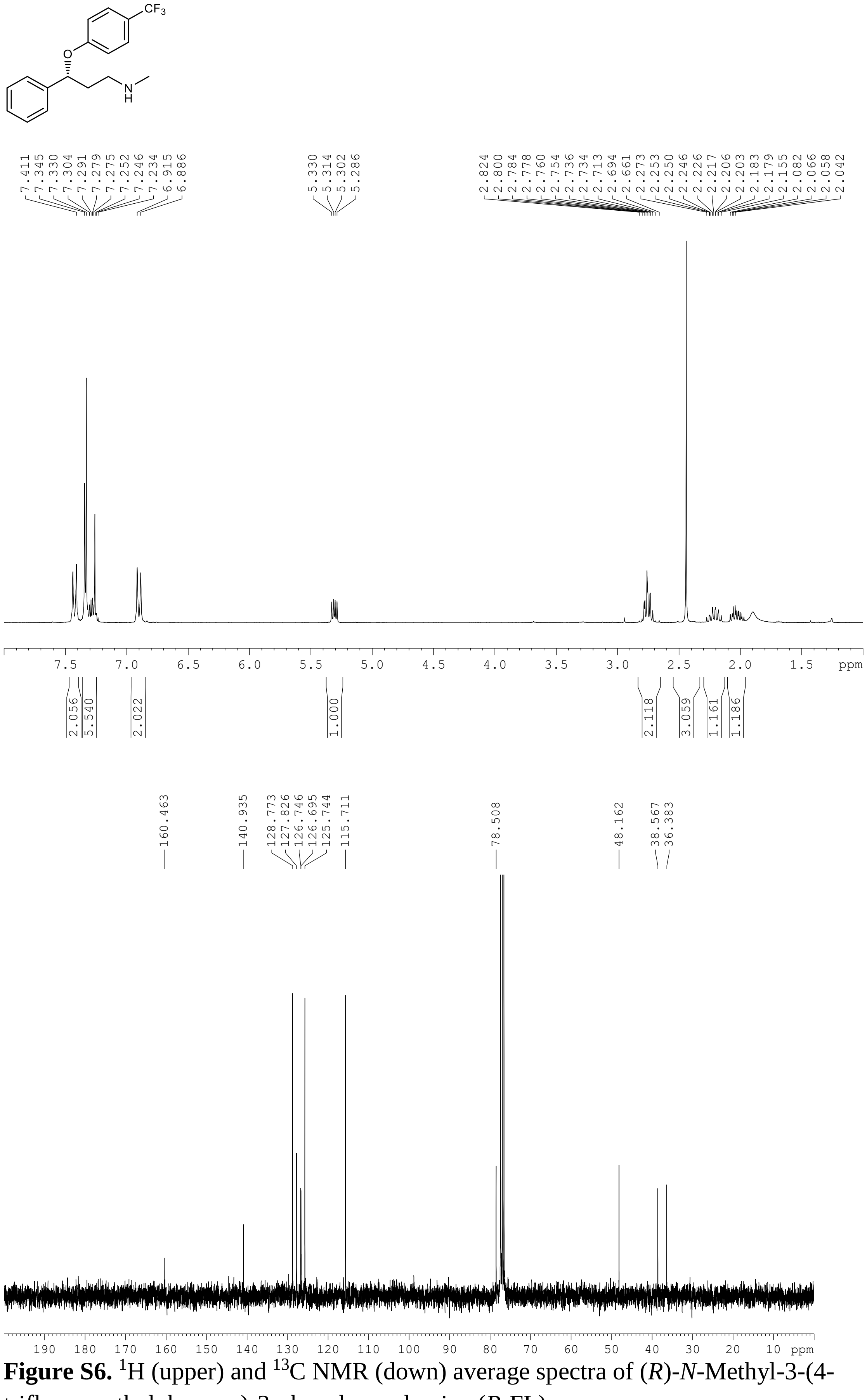

Figure S6. ^{1}H (upper) and ^{13}C NMR (down) average spectra of (*R*)-*N*-Methyl-3-(4-trifluoromethylphenoxy)-3-phenylpropylamine (*R*-FL)

Table S1. Chromatographic parameters (retention time, R_t; resolution separation, R_s) obtained using different mobile phase modifiers (MeOH; EtOH), additives (AAC; FA) and flows.

Mobile Phase					Rt (*S*/*R*)		R_s	
Organic phase (%)	Aqueuous phase (%)	Buffer concentration (mM AAC)	Acidifier (% FA)	Flow mL min^{-1}	FL	NFL	FL	NFL
80 MeOH	20	1	0.005	0.1	17.63/19.11	17.16/17.90	0.89	0.64
				0.06	35.37/38.25	34.54/35.93	1.35	0.8
80 MeOH	20	4	0.005	0.1	25.24/27.29	24.23/25.43	0.94	0.86
				0,06	50.89/55.07	48.94/50.89	1.19	0.72
80 MeOH	20	10	0.005	0.1	16.97/18.00	16.05/16.7		n.s.
				0.06	--	--	--	--
70 MeOH	30	1	0.005	0.1	15.58/16.70	14.41/15.95	1.1	0.71
				0.06	31.03/33.33	30.54/31.75	1.12	0.71
70 MeOH	30	4	0.005	0.1	24.22/25.99	23.57/24.59	0.96	0.81
				0,06	48.38/52.28	47.36/49.31	1.27	0.76
70 MeOH	30	10	0.005	0.1	16.60/17.81	16.14/16.60	--	ns
				0.06	--	--	--	--
50 EtOH	50	1		0.06	68.73/75.95	63.06/66.96	1.85	1.02
70 EtOH	30	1		0.06	78.08/86.20	71.61/76.81	1.41	1.07
80 EtOH	20	1		0.06	>90	>90		
92.5 EtOH	7.5	1		0.06	>90	>90		
70 EtOH	30	1	0.005	0.06	25.15/27.5	23.76/25.15	1.21	0.85
70 EtOH	30	4	0.005	0.06	35.37/39.55	33.79/36.12	1.41	1

Table S2. Performance data: linearity, R^2; instrumental and method limits of detection and quantification; resolution (R_s).

Compound	Enantiomer	Instrumental parameters							River water		Activated sludge	
		Linearity range (µg L^{-1})	R^2	$IDL_{s/n}$ (µg L^{-1})	$IQL_{s/n}$ (µg L^{-1})	R_s			MDL_{CALC} (ng L^{-1})	MQL_{CALC} (ng L^{-1})	MDL_{CALC} (ng L^{-1})	MQL_{CALC} (ng L^{-1})
						1 µg L^{-1}	50 µg L^{-1}	100 µg L^{-1}				
FL	*S* (+)	0.5-100	0.992	0.12	0.5	1.4 ± 0.2	1.4 ± 0.3	1.5 ± 0.4	1.2	4.6	0.8	3.1
FL	*R* (-)	0.5-100	0.991	0.12	0.5				1.3	5.1	0.5	2.0
NFL	*S* (+)	0.5-100	0.994	0.12	0.5	1.0 ± 0.4	1.1 ± 0.1	1.1 ± 0.1	1.3	5.1	0.4	1.7
NFL	*R* (-)	0.5-100	0.996	0.12	0.5				1.2	4.7	0.7	2.7

Table S3. Performance data: retention time, Rt; inter and intra-day repeatability.

Compound	Enantiomer	Rt (min)	Instrumental parameters												Method parameters											
			Intra-day (RSD%, n=3)									Inter-day (RSD%, n=3)			Intra-day (RSD%, n=3)									Inter-day (RSD%, n=3)		
			5 µg L^{-1}			25 µg L^{-1}			250 µg L^{-1}			5 µg L^{-1}	25 µg L^{-1}	250 µg L^{-1}	5 µg L^{-1}			25 µg L^{-1}			250µg L^{-1}			5 µg L^{-1}	25 µg L^{-1}	250 µg L^{-1}
			D1	D2	D3	D1	D2	D3	D1	D2	D3				D1	D2	D3	D1	D2	D3	D1	D2	D3			
FL	S (+)	35.4	6.5	9.3	13.2	7.0	5.4	6.1	2.1	3.7	2.6	11.0	6.1	2.6	3.6	4.1	1.6	2.4	2.9	8.7	3.0	0.7	1.7	2.5	3.4	3.5
FL	R (-)	39.6	12.8	2.5	10.6	6.0	8.3	7.4	1.2	1.8	1.3	14.4	7.4	1.3	14.6	5.3	7.5	2.2	3.9	7.3	2.8	4.4	3.3	4.8	8.0	4.3
NFL	S (+)	33.8	6.5	5.4	10.1	5.5	4.4	4.2	1.0	2.1	2.0	8.4	4.2	2.0	19.8	17.0	17.6	12.2	12.3	12.2	2.3	2.2	2.1	3.2	3.9	2.7
NFL	R (-)	36.1	6.2	6.3	1.1	3.7	6.0	5.8	0.7	1.0	1.5	7.0	5.8	1.5	4.6	15.2	8.0	14.6	12.5	16.3	2.4	1.9	0.4	11.1	4.4	2.6

D1, D2, D3: days 1, 2 and 3.

Table S4. Transformation of FL and formation of NFL in river water simulating microcosms under dark abiotic (DAR), dark biotic (DBR), light abiotic (LAR) and light biotic (LBR) conditions (n.a. not analysed; n.d. not detected).

Time [days]	DAR					
	FL			NFL		
	Concentration [μg L^{-1}]		EF	Concentration [μg L^{-1}]		EF
	S-FL	R-FL		S-NFL	R-NFL	
1	0.478 ± 0.079	0.481 ± 0.082	0.499	n.d.	n.d.	n.d.
2	0.535 ± 0.097	0.529 ± 0.088	0.502	n.d.	n.d.	n.d.
3	0.573 ± 0.052	0.566 ± 0.061	0.504	n.d.	n.d.	n.d.
4	0.449 ± 0.036	0.437 ± 0.027	0.507	n.d.	n.d.	n.d.
5	0.479 ± 0.096	0.480 ± 0.102	0.480	n.d.	0.002 ± 0.000	0.000
6	n.a.	n.a.	n.a.	n.a.	n.a.	n.a.
7	n.a.	n.a.	n.a.	n.a.	n.a.	n.a.
8	0.460 ± 0.079	0.443 ± 0.073	0.509	n.d.	n.d.	n.d.
9	0.459 ± 0.043	0.466 ± 0.050	0.497	n.d.	n.d.	n.d.
10	0.428 ± 0.146	0.417 ± 0.138	0.505	n.d.	n.d.	n.d.
11	0.431 ± 0.196	0.387 ± 0.174	0.526	n.d.	n.d.	n.d.
12	0.428 ± 0.142	0.420 ± 0.141	0.509	n.d.	n.d.	n.d.
13	n.a.	n.a.	n.a.	n.a.	n.a.	n.a.
14	n.a.	n.a.	n.a.	n.a.	n.a.	n.a.
15	0.405 ± 0.077	0.386 ± 0.093	0.497	n.d.	n.d.	n.d.
16	0.470 ± 0.117	0.455 ± 0.117	0.509	n.d.	n.d.	n.d.

Time [days]	DBR					
	FL			NFL		
	Concentration [μg L^{-1}]		EF	Concentration [μg L^{-1}]		EF
	S-FL	R-FL		S-NFL	R-NFL	
1	0.406 ± 0.007	0.397 ± 0.004	0.506	n.d.	0.002 ± 0.001	0.000
2	0.459 ± 0.058	0.525 ± 0.081	0.468	n.d.	n.d.	n.d.
3	0.624 ± 0.084	0.634 ± 0.081	0.496	0.001 ± 0.000	0.002 ± 0.001	0.333
4	0.497 ± 0.076	0.491 ± 0.069	0.503	n.d.	n.d.	n.d.
5	0.537 ± 0.139	0.547 ± 0.136	0.495	0.001 ± 0.000	0.002 ± 0.001	0.417
6	n.a.	n.a.	n.a.	n.a.	n.a.	n.a.
7	n.a.	n.a.	n.a.	n.a.	n.a.	n.a.
8	0.317 ± 0.004	0.431 ± 0.197	0.441	n.d.	n.d.	n.d.
9	0.317 ± 0.055	0.319 ± 0.071	0.499	n.d.	n.d.	n.d.
10	0.299 ± 0.011	0.291 ± 0.012	0.507	n.d.	n.d.	n.d.
11	0.283 ± 0.061	0.287 ± 0.047	0.495	n.d.	n.d.	n.d.
12	0.251 ± 0.066	0.257 ± 0.035	0.490	n.d.	n.d.	n.d.
13	n.a.	n.a.	n.a.	n.a.	n.a.	n.a.
14	n.a.	n.a.	n.a.	n.a.	n.a.	n.a.
15	0.191 ± 0.040	0.162 ± 0.026	0.538	n.d.	n.d.	n.d.
16	0.161 ± 0.057	0.127 ± 0.034	0.554	n.d.	n.d.	n.d.

Time [days]	LAR					
	FL			NFL		
	Concentration [μg L^{-1}]		EF	Concentration [μg L^{-1}]		EF
	S-FL	R-FL		S-NFL	R-NFL	
1	0.485 ± 0.135	0.489 ± 0.125	0.497	n.d.	n.d.	n.d.
2	0.397 ± 0.008	0.417 ± 0.006	0.488	n.d.	0.001 ± 0.000	0.000
3	0.489 ± 0.042	0.506 ± 0.049	0.491	0.002 ± 0.001	0.004 ± 0.001	0.358
4	0.372 ± 0.047	0.377 ± 0.059	0.498	0.001 ± 0.000	0.003 ± 0.000	0.250
5	0.287 ± 0.029	0.275 ± 0.038	0.511	n.d.	0.002 ± 0.001	0.000
6	n.a.	n.a.	n.a.	n.a.	n.a.	n.a.
7	n.a.	n.a.	n.a.	n.a.	n.a.	n.a.
8	0.275 ± 0.039	0.265 ± 0.027	0.508	n.d.	0.001 ± 0.001	0.000
9	0.227 ± 0.022	0.216 ± 0.028	0.513	n.d.	0.002 ± 0.001	0.000
10	0.219 ± 0.067	0.209 ± 0.058	0.511	n.d.	0.002 ± 0.000	0.000
11	0.243 ± 0.056	0.233 ± 0.064	0.512	n.d.	0.003 ± 0.000	0.000

12	0.187 ± 0.047	0.182 ± 0.045	0.487	0.002 ± 0.001	0.003 ± 0.001	0.225
13	n.a.	n.a.	n.a.	n.a.	n.a.	n.a.
14	n.a.	n.a.	n.a.	n.a.	n.a.	n.a.
15	0.080 ± 0.019	0.072 ± 0.022	0.531	n.d.	0.002 ± 0.001	0.000
16	0.113 ± 0.039	0.101 ± 0.043	0.535	0.001 ± 0.000	0.004 ± 0.001	0.225

Time [days]	LBR					
	FL			NFL		
	Concentration [μg L^{-1}]		EF	Concentration [μg L^{-1}]		EF
	S-FL	R-FL		S-NFL	R-NFL	
1	0.467 ± 0.059	0.481 ± 0.058	0.493	n.d.	0.001± 0.000	0.000
2	0.329 ± 0.014	0.338 ± 0.027	0.493	n.d.	0.002 ± 0.000	0.000
3	0.333 ± 0.046	0.335 ± 0.053	0.498	0.001 ± 0.000	0.002 ± 0.001	0.292
4	0.275 ± 0.098	0.273 ± 0.096	0.502	n.d.	n.d.	n.d.
5	0.278 ± 0.026	0.269 ± 0.024	0.508	0.001 ± 0.000	0.003 ± 0.001	0.292
6	n.a.	n.a.	n.a.	n.a.	n.a.	n.a.
7	n.a.	n.a.	n.a.	n.a.	n.a.	n.a.
8	0.091 ± 0.043	0.085 ± 0.061	0.537	n.d.	n.d.	n.d.
9	0.083 ± 0.024	0.088 ± 0.059	0.512	n.d.	n.d.	n.d.
10	0.034 ± 0.019	0.043 ± 0.044	0.497	n.d.	n.d.	n.d.
11	0.051 ± 0.011	0.055 ± 0.026	0.498	n.d.	n.d.	n.d.
12	0.030 ± 0.011	0.034 ± 0.016	0.491	n.d.	n.d.	n.d.
13	n.a.	n.a.	n.a.	n.a.	n.a.	n.a.
14	n.a.	n.a.	n.a.	n.a.	n.a.	n.a.
15	0.013 ± 0.009	0.021 ± 0.022	0.525	n.d.	n.d.	n.d.
16	0.007 ± 0.006	0.016 ± 0.001	0.467	n.d.	n.d.	n.d.

Table S5. Transformation of FL and formation of NFL in activated sludge simulating microcosms under dark biotic (DBR) at two spiked levels of FL (10 and 100 µg L^{-1}) and 'no spike' (n.d. not detected).

Time [days]	10 µg L^{-1} microcosm					
	FL			NFL		
	Concentration [µg L^{-1}]		EF	Concentration [µg L^{-1}]		EF
	S-FL	R-FL		S-NFL	R-NFL	
0	5.089 ± 0.305	5.490 ± 1.033	0.484	0.127 ± 0.015	0.035 ± 0.009	0.784
0.5	2.927 ± 0.216	2.879 ± 0.167	0.504	0.057 ± 0.006	0.029 ± 0.001	0.663
1	2.201 ± 0.128	4.135 ± 0.030	0.347	n.d.	0.077 ± 0.004	0.000
1.5	2.514 ± 0.002	2.768 ± 0.289	0.499	0.010 ± 0.000	0.012 ± 0.013	0.887
2	2.237 ± 0.124	2.867 ± 0.533	0.480	0.151 ± 0.068	0.050 ± 0.011	0.750
3	2.502 ± 0.251	2.875 ± 0.437	0.499	0.222 ± 0.038	0.043 ± 0.022	0.839
5	2.265 ± 0.027	3.019 ± 0.170	0.429	0.384 ± 0.053	0.070 ± 0.016	0.810
8	1.588 ± 0.529	1.811 ± 0.055	0.461	0.315 ± 0.016	0.055 ± 0.019	0.856
12	1.075 ± 0.556	2.949 ± 0.299	0.263	0.562 ± 0.219	0.126 ± 0.035	0.749
24	1.331 ± 0.000	3.001 ± 0.691	0.260	0.510 ± 0.059	0.182 ± 0.000	0.752

Time [days]	100 µg L^{-1} microcosm					
	FL			NFL		
	Concentration [µg L^{-1}]		EF	Concentration [µg L^{-1}]		EF
	S-FL	R-FL		S-NFL	R-NFL	
0	49.932 ± 2.414	43.555 ± 1.748	0.531	0.553 ± 0.146	0.109 ± 0.023	0.833
0.5	49.186 ± 3.891	46.115 ± 9.344	0.519	0.538 ± 0.149	0.132 ± 0.015	0.826
1	42.865 ± 3.091	40.297 ± 2.172	0.515	0.737 ± 0.154	0.128 ± 0.038	0.858
1.5	42.894 ± 1.940	40.183 ± 0.741	0.516	0.981 ± 0.052	0.146 ± 0.039	0.872
2	47.786 ± 3.348	45.913 ± 5.113	0.511	1.890 ± 0.622	0.428 ± 0.019	0.848
3	34.498 ± 4.717	37.624 ± 3.543	0.477	2.515 ± 1.049	0.429 ± 0.164	0.852
5	26.024 ± 0.982	35.269 ± 1.956	0.431	3.535 ± 1.300	0.616 ± 0.227	0.851
8	23.630 ± 0.614	33.823 ± 4.145	0.410	3.844 ± 1.117	0.710 ± 0.193	0.843
12	22.875 ± 1.267	46.372 ± 4.755	0.326	5.346 ± 0.464	0.859 ± 0.069	0.864
24	9.842 ± 0.916	26.774 ± 0.193	0.257	5.046 ± 0.415	1.302 ± 0.066	0.794

Time [days]	'No spike' microcosm					
	FL			NFL		
	Concentration [µg L^{-1}]		EF	Concentration [µg L^{-1}]		EF
	S-FL	R-FL		S-NFL	R-NFL	
0	0.249 ± 0.003	0.241 ± 0.005	0.509	0.007 ± 0.000	0.002 ± 0.000	0.789
0.5	0.739 ± 0.008	0.638 ± 0.060	0.536	0.038 ± 0.001	0.032 ± 0.007	0.549
1	0.227 ± 0.004	0.232 ± 0.008	0.495	0.007 ± 0.001	0.002 ± 0.002	0.795
1.5	0.097 ± 0.001	0.097 ± 0.003	0.500	0.005 ± 0.001	0.001 ± 0.001	0.843
2	0.493 ± 0.014	0.623 ± 0.003	0.442	0.068 ± 0.029	0.031 ± 0.025	0.720
3	0.596 ± 0.041	0.627 ± 0.056	0.488	0.030 ± 0.004	0.007 ± 0.006	0.810
5	0.972 ± 0.043	1.403 ± 0.068	0.409	0.124 ± 0.036	0.055 ± 0.021	0.706
8	1.213 ± 0.103	2.281 ± 0.151	0.347	0.197 ± 0.008	0.028 ± 0.019	0.804
12	0.681 ± 0.091	1.072 ± 0.251	0.364	0.340 ± 0.050	0.017 ± 0.000	0.947
24	0.518 ± 0.100	0.553 ± 0.087	0.503	0.228 ± 0.049	0.043 ± 0.020	0.849

Table S6. Raw data of Daphtoxkit F Magna. 48 h immobile *D. magna* for each toxicant concentration.

(*R*)-FL

Concentration	Average effect	Standard deviation	Total number replicates (Rep)	N immobiles			
				Rep1	Rep2	Rep3	Rep4
0	0	0	4	0	0	0	0
0.5	0	0	4	0	0	0	0
1.5	0.25	0.5	4	1	0	0	0
5	3.25	0.5	4	3	3	4	3
16	5	0	4	5	5	5	5
50	5	0	4	5	5	5	5

(*S*)-FL

Concentration	Average effect	Standard deviation	Total number replicates (Rep)	N immobile			
				Rep1	Rep2	Rep3	Rep4
0	0	0	4	0	0	0	0
0.5	0.25	0.5	4	0	0	0	1
1.5	1	0	4	1	1	1	1
5	3.25	0.5	4	3	3	3	4
16	5	0	4	5	5	5	5
50	5	0	4	5	5	5	5

(*R*)-NFL

Concentration	Average effect	Standard deviation	Total number replicates (Rep)	N immobile			
				Rep1	Rep2	Rep3	Rep4
0	0	0	4	0	0	0	0
0.5	0.25	0.5	4	0	1	0	0
1.5	0.5	0.6	4	0	1	1	0
5	4.25	0.5	4	5	4	4	4
16	5	0	4	5	5	5	5
50	5	0	4	5	5	5	5

(*S*)-NFL

Concentration	Average effect	Standard deviation	Total number replicates (Rep)	N immobile			
				Rep1	Rep2	Rep3	Rep4
0	0	0	4	0	0	0	0
0.5	0.25	0.5	4	0	0	0	1
1.5	1	0.8	4	1	1	2	0
5	4	0.8	4	4	4	3	5
16	5	0	4	5	5	5	5
50	5	0	4	5	5	5	5

Table S7. *R*-FL Range

Results – Optical Density

Conc.	Time	Replicate		Mean	Std. dev.	CV%
		1	2			
	t0	0.630	0.631	0.631	0.001	0.11%
Control	t24	0.223	0.265	0.244	0.030	12.17%
	t0	0.650	0.644	0.647	0.004	0.66%
0.005	t24	0.240	0.257	0.249	0.012	4.84%
	t0	0.642	0.638	0.640	0.003	0.44%
0.050	t24	0.197	0.352	0.275	0.110	39.93%
	t0	0.630	0.632	0.631	0.001	0.22%
0.500	t24	0.220	0.260	0.240	0.028	11.79%
	t0	0.646	0.647	0.647	0.001	0.11%
5.000	t24	0.402	0.427	0.415	0.018	4.26%
	t0	0.646	0.647	0.647	0.001	0.11%
50.000	t24	0.644	0.636	0.640	0.006	0.88%

Summary of Results

Conc.	Statistics	Time (hours)	
		0	24
Control	Mean	0.631	0.244
	CV%	0.11%	12.17%
0.005	Mean	0.647	0.249
	CV%	0.66%	4.84%
0.050	Mean	0.640	0.275
	CV%	0.44%	39.93%
0.500	Mean	0.631	0.240
	CV%	0.22%	11.79%
5.000	Mean	0.647	0.415
	CV%	0.11%	4.26%
50.000	Mean	0.647	0.640
	CV%	0.11%	0.88%

Percentage Inhibition Computation

Conc.	0	100%	% I
Control	0.244	0.387	0.000
0.005			-3.105
0.050			5.433
0.500			-1.164
5.000			39.974
50.000			98.318

Concentration vs. Percent Inhibition

Log Conc.	I%	Conc.
-2.301	-3.10	0.005
-1.301	5.43	0.050
-0.301	-1.16	0.500
0.699	39.97	5.000
1.699	98.32	50.000

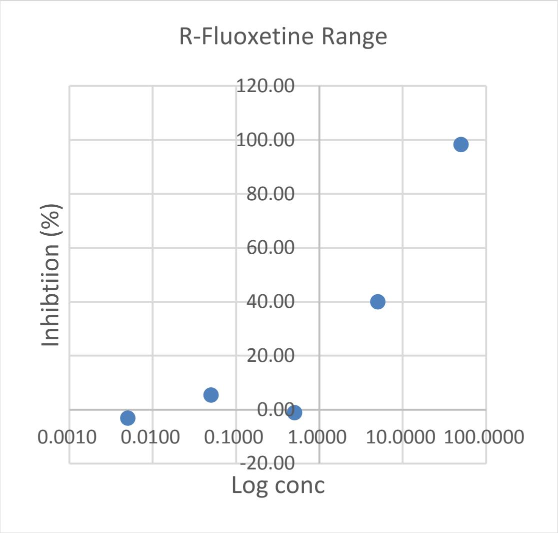

Effect Concentration Results

**log x =**	**-1.055**
**24hEC10 =**	**0.088**
**log x =**	**-0.633**
**24hEC20 =**	**0.233**
**log x =**	**0.630**
**24hEC50 =**	**4.262**
**log x =**	**1.473**
**24hEC70 =**	**29.591**
**log x =**	**2.315**
**24hEC90 =**	**205.456**

Lower 95%	Upper 95%
-0.407	70.482

Summary Output

Regression Statistics	
Multiple R	0.872
R Square	0.760
Adjusted R Square	0.680
Standard Error	24.359
Observations	5

ANOVA

	df	*SS*	*MS*	*F*	*Significance F*
Regression	1	5635.250	5635.250	9.497	0.054
Residual	3	1780.042	593.347		
Total	4	7415.292			

	Coefficients	*Standard Error*	*t Stat*	*P-value*	*Lower 95%*	*Upper 95%*	*Lower 95.0%*	*Upper 95.0%*
Intercept	35.037	11.138	3.146	0.051	-0.407	70.482	-0.407	70.482
X Variable 1	23.739	7.703	3.082	0.054	-0.775	48.253	-0.775	48.253

Table S8. *R*-FL Definitive

Results – Optical Density

Conc.	Time	Replicate		Mean	Std. dev.	CV%
		1	2			
	t0	0.701	0.698	0.700	0.002	0.30%
0.000	t24	0.276	0.239	0.258	0.026	10.16%
	t0	0.705	0.687	0.696	0.013	1.83%
0.029	t24	0.293	0.260	0.277	0.023	8.44%
	t0	0.701	0.698	0.700	0.002	0.30%
0.086	t24	0.173	0.271	0.222	0.069	31.21%
	t0	0.696	0.693	0.695	0.002	0.31%
2.874	t24	0.665	0.662	0.664	0.002	0.32%
	t0	0.702	0.701	0.702	0.001	0.10%
24.098	t24	0.638	0.690	0.664	0.037	5.54%
	t0	0.704	0.703	0.704	0.001	0.10%
38.556	t24	0.696	0.646	0.671	0.035	5.27%
	t0	0.697	0.709	0.703	0.008	1.21%
79.522	t24	0.688	0.636	0.662	0.037	5.55%
	t0	0.708	0.715	0.712	0.005	0.70%
120.488	t24	0.711	0.717	0.714	0.004	0.59%

Summary of Results

Conc.	Statistics	Time (hours)	
		0	24
0.00	Mean	0.700	0.258
	CV%	0.30%	10.16%
0.029	Mean	0.696	0.277
	CV%	1.83%	8.44%
0.086	Mean	0.700	0.222
	CV%	0.30%	31.21%
2.874	Mean	0.695	0.664
	CV%	0.31%	0.32%
24.098	Mean	0.702	0.664
	CV%	0.10%	5.54%
38.556	Mean	0.704	0.671
	CV%	0.10%	5.27%
79.522	Mean	0.703	0.662
	CV%	1.21%	5.55%
120.488	Mean	0.712	0.714
	CV%	0.70%	0.59%

Percentage Inhibition Computation

Conc.	0	100%	% I
0.00	0.258	0.442	0.000
0.029			5.090
0.086			-8.032
2.874			92.986
24.098			91.516
38.556			92.647
79.522			90.724
120.488			100.566

Concentration vs. Percent Inhibition

Log Conc.	I%	Conc.
-1.538	5.09	0.029
-1.066	-8.03	0.086
0.458	92.99	2.874
1.382	91.52	24.098
1.586	92.65	38.556
1.900	90.72	79.522
2.081	100.57	120.488

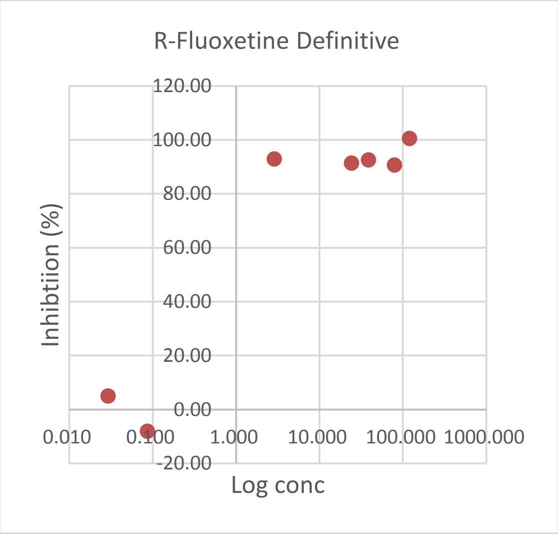

Effect Concentration Results

**log x =**	**-1.219**
**24hEC10 =**	**0.061**
**log x =**	**-0.882**
**24hEC20 =**	**0.132**
**log x =**	**0.130**
**24hEC50 =**	**1.348**
**log x =**	**0.804**
**24hEC70 =**	**6.361**
**log x =**	**1.479**
**24hEC90 =**	**30.009**

Lower 95%	Upper 95%
25.199	67.091

Summary Output

Regression Statistics	
Multiple R	0.927
R Square	0.859
Adjusted R Square	0.831
Standard Error	19.221
Observations	7

ANOVA

	df	*SS*	*MS*	*F*	*Significance F*
Regression	1	11237.216	11237.216	30.418	0.003
Residual	5	1847.140	369.428		
Total	6	13084.356			

	Coefficients	*Standard Error*	*t Stat*	*P-value*	*Lower 95%*	*Upper 95%*	*Lower 95.0%*	*Upper 95.0%*
Intercept	46.145	8.148	5.663	0.002	25.199	67.091	25.199	67.091
X Variable 1	29.653	5.377	5.515	0.003	15.832	43.475	15.832	43.475

Table S9. *S*-FL Range

Results – Optical Density

Conc.	Time	Replicate		Mean	Std. dev.	CV%
		1	2			
	t0	0.681	0.671	0.676	0.007	1.05%
Control	t24	0.028	0.271	0.149	0.172	115.11%
	t0	0.653	0.668	0.661	0.011	1.61%
0.005	t24	0.204	0.302	0.253	0.069	27.39%
	t0	0.661	0.668	0.665	0.005	0.74%
0.050	t24	0.204	0.302	0.253	0.069	27.39%
	t0	0.660	0.681	0.671	0.015	2.21%
0.500	t24	0.281	0.286	0.284	0.004	1.25%
	t0	0.665	0.652	0.659	0.009	1.40%
5.000	t24	0.223	0.316	0.270	0.066	24.40%
	t0	0.662	0.669	0.666	0.005	0.74%
50.000	t24	0.431	0.454	0.443	0.016	3.68%

Summary of Results

Conc.	Statistics	Time (hours)	
		0	24
Control	Mean	0.676	0.149
	CV%	1.05%	115.11%
0.005	Mean	0.661	0.253
	CV%	1.61%	27.39%
0.050	Mean	0.665	0.253
	CV%	0.74%	27.39%
0.500	Mean	0.671	0.284
	CV%	2.21%	1.25%
5.000	Mean	0.659	0.270
	CV%	1.40%	24.40%
50.000	Mean	0.666	0.443
	CV%	0.74%	3.68%

Percentage Inhibition Computation

Conc.	0	100%	% I
Control	0.149	0.527	0.000
0.005			22.617
0.050			21.857
0.500			26.510
5.000			26.130
50.000			57.653

Concentration vs. Percent Inhibition

Log Conc.	I%	Conc
-2.301	22.62	0.005
-1.301	21.86	0.050
-0.301	26.51	0.500
0.699	26.13	5.000
1.699	57.65	50.000

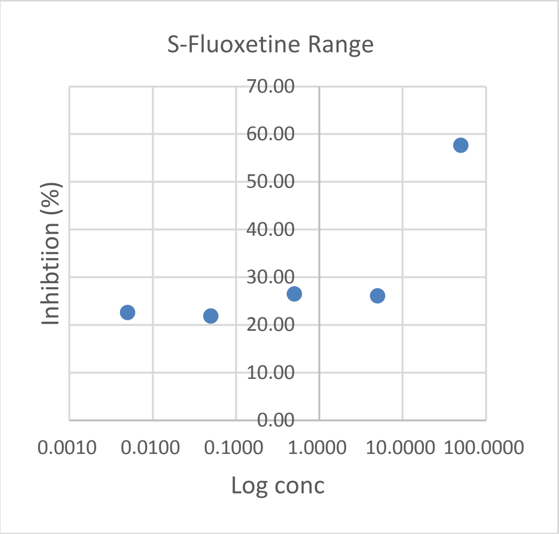

Effect Concentration Results

**log x =**	**-3.119**
**24hEC10 =**	**0.001**
**log x =**	**-1.774**
**24hEC20 =**	**0.017**
**log x =**	**2.261**
**24hEC50 =**	**181.290**
**log x =**	**4.951**
**24hEC70 =**	**88210.189**
**log x =**	**7.641**
**24hEC90 =**	**42920478.224**

Lower 95%	Upper 95%
17.353	49.029

Summary Output

Regression Statistics	
Multiple R	0.780
R Square	0.609
Adjusted R Square	0.478
Standard Error	10.884
Observations	5

ANOVA

	df	*SS*	*MS*	*F*	*Significance F*
Regression	1	552.716	552.716	4.666	0.120
Residual	3	355.399	118.466		
Total	4	908.114			

	Coefficients	*Standard Error*	*t Stat*	*P-value*	*Lower 95%*	*Upper 95%*	*Lower 95.0%*	*Upper 95.0%*
Intercept	33.191	4.977	6.669	0.007	17.353	49.029	17.353	49.029
X Variable 1	7.434	3.442	2.160	0.120	-3.519	18.388	-3.519	18.388

Table S10. *S*-FL Definitive

Results – Optical Density

**Conc.**	**Time**	**Replicate**		**Mean**	**Std. dev.**	**CV%**
		**1**	**2**			
	t0	0.649	0.663	0.656	0.010	1.51%
0.000	t24	0.174	0.208	0.191	0.024	12.59%
	t0	0.641	0.643	0.642	0.001	0.22%
0.104	t24	0.183	0.155	0.169	0.020	11.72%
	t0	0.645	0.645	0.645	0.000	0.00%
0.313	t24	0.231	0.168	0.200	0.045	22.33%
	t0	0.646	0.648	0.647	0.001	0.22%
0.535	t24	0.187	0.178	0.183	0.006	3.49%
	t0	0.649	0.658	0.654	0.006	0.97%
10.446	t24	0.189	0.255	0.222	0.047	21.02%
	t0	0.645	0.649	0.647	0.003	0.44%
20.892	t24	0.289	0.283	0.286	0.004	1.48%
	t0	0.649	0.656	0.653	0.005	0.76%
68.945	t24	0.542	0.516	0.529	0.018	3.48%
	t0	0.659	0.656	0.658	0.002	0.32%
104.462	t24	0.552	0.606	0.579	0.038	6.59%

Summary of Results

**Conc.**	**Statistics**	**Time (hours)**	
		**0**	**24**
0.00	Mean	0.656	0.191
	CV%	1.51%	12.59%
0.104	Mean	0.642	0.169
	CV%	0.22%	11.72%
0.313	Mean	0.645	0.200
	CV%	0.00%	22.33%
0.535	Mean	0.647	0.183
	CV%	0.22%	3.49%
10.446	Mean	0.654	0.222
	CV%	0.97%	21.02%
20.892	Mean	0.647	0.286
	CV%	0.44%	1.48%
68.945	Mean	0.653	0.529
	CV%	0.76%	3.48%
104.462	Mean	0.658	0.579
	CV%	0.32%	6.59%

Percentage Inhibition Computation

**Conc.**	**0**	**100%**	**% I**
0.00	0.191	0.465	0.000
0.104			-1.720
0.313			4.194
0.535			0.108
10.446			7.204
20.892			22.366
68.945			73.441
104.462			83.118

Concentration vs. Percent Inhibition

Log Conc.	I%	Conc.
-0.983	-1.72	0.104
-0.504	4.19	0.313
-0.272	0.11	0.535
1.019	7.20	10.446
1.320	22.37	20.892
1.839	73.44	68.945
2.019	83.12	104.462

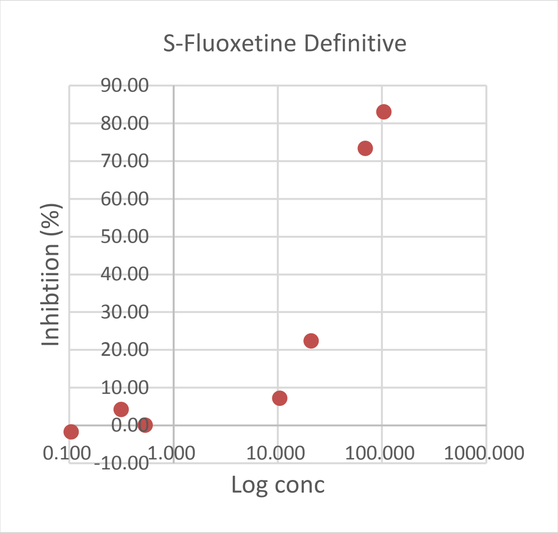

Effect Concentration Results

**log x =**	**-0.039**
**24hEC10 =**	**0.914**
**log x =**	**0.358**
**24hEC20 =**	**2.276**
**log x =**	**1.549**
**24hEC50 =**	**35.227**
**log x =**	**2.343**
**24hEC70 =**	**218.753**
**log x =**	**3.137**
**24hEC90 =**	**1358.412**

Lower 95%	Upper 95%
-12.741	34.722

Summary Output

Regression Statistics	
Multiple R	0.843
R Square	0.710
Adjusted R Square	0.652
Standard Error	21.238
Observations	7

ANOVA

	df	*SS*	*MS*	*F*	*Significance F*
Regression	1	5532.187	5532.187	12.264	0.017
Residual	5	2255.366	451.073		
Total	6	7787.553			

	Coefficients	*Standard Error*	*t Stat*	*P-value*	*Lower 95%*	*Upper 95%*	*Lower 95.0%*	*Upper 95.0%*
Intercept	10.991	9.232	1.190	0.287	-12.741	34.722	-12.741	34.722
X Variable 1	25.190	7.193	3.502	0.017	6.700	43.680	6.700	43.680

Table S11. *R*-NFL Range

Results – Optical Density

Conc.	Time	Replicate		Mean	Std. dev.	CV%
		1	2			
	t0	0.655	0.655	0.655	0.000	0.00%
Control	t24	0.215	0.283	0.249	0.048	19.31%
	t0	0.646	0.646	0.646	0.000	0.00%
0.005	t24	0.372	0.194	0.283	0.126	44.48%
	t0	0.655	0.667	0.661	0.008	1.28%
0.050	t24	0.225	0.226	0.226	0.001	0.31%
	t0	0.657	0.654	0.656	0.002	0.32%
0.500	t24	0.440	0.468	0.454	0.020	4.36%
	t0	0.637	0.651	0.644	0.010	1.54%
5.000	t24	0.646	0.651	0.649	0.004	0.55%
	t0	0.693	0.675	0.684	0.013	1.86%
50.000	t24	0.716	0.708	0.712	0.006	0.79%

Summary of Results

Conc.	Statistics	Time (hours)	
		0	24
Control	Mean	0.655	0.249
	CV%	0.00%	19.31%
0.005	Mean	0.646	0.283
	CV%	0.00%	44.48%
0.050	Mean	0.661	0.226
	CV%	1.28%	0.31%
0.500	Mean	0.656	0.454
	CV%	0.32%	4.36%
5.000	Mean	0.644	0.649
	CV%	1.54%	0.55%
50.000	Mean	0.684	0.712
	CV%	1.86%	0.79%

Percentage Inhibition Computation

Conc.	0	100%	% I
Control	0.249	0.406	0.000
0.005			10.591
0.050			-7.266
0.500			50.369
5.000			101.108
50.000			106.897

Concentration vs. Percent Inhibition

Log Conc.	I%	Conc.
-2.301	10.59	0.005
-1.301	-7.27	0.050
-0.301	50.37	0.500
0.699	101.11	5.000
1.699	106.90	50.000

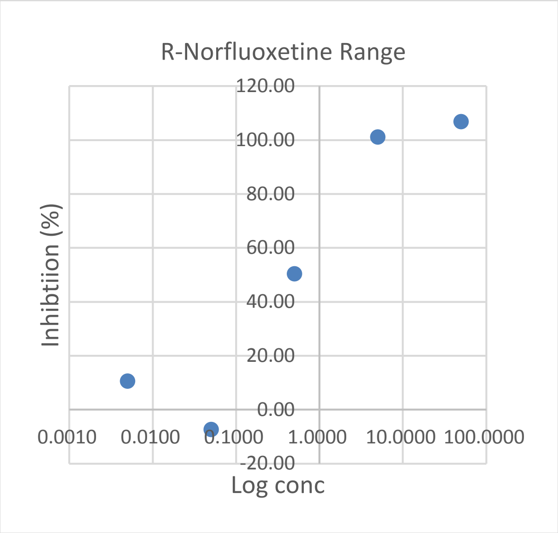

Effect Concentration Results

**log x =**	**-1.708**
**24hEC10 =**	**0.020**
**log x =**	**-1.375**
**24hEC20 =**	**0.042**
**log x =**	**-0.379**
**24hEC50 =**	**0.418**
**log x =**	**0.286**
**24hEC70 =**	**1.929**
**log x =**	**0.950**
**24hEC90 =**	**8.895**

Lower 95%	Upper 95%
27.845	94.955

Summary Output

Regression Statistics	
Multiple R	0.922
R Square	0.850
Adjusted R Square	0.800
Standard Error	23.060
Observations	5

ANOVA

	df	*SS*	*MS*	*F*	*Significance F*
Regression	1	9059.210	9059.210	17.036	0.026
Residual	3	1595.289	531.763		
Total	4	10654.499			

	Coefficients	*Standard Error*	*t Stat*	*P-value*	*Lower 95%*	*Upper 95%*	*Lower 95.0%*	*Upper 95.0%*
Intercept	61.400	10.544	5.823	0.010	27.845	94.955	27.845	94.955
X Variable 1	30.099	7.292	4.127	0.026	6.891	53.306	6.891	53.306

Table S12. *R*-NFL Definitive

Results – Optical Density

Conc.	Time	Replicate		Mean	Std. dev.	CV%
		1	2			
	t0	0.662	0.675	0.669	0.009	1.38%
0.000	t24	0.196	0.206	0.201	0.007	3.52%
	t0	0.662	0.660	0.661	0.001	0.21%
0.220	t24	0.198	0.148	0.173	0.035	20.44%
	t0	0.667	0.667	0.667	0.000	0.00%
0.661	t24	0.136	0.154	0.145	0.013	8.78%
	t0	0.668	0.686	0.677	0.013	1.88%
22.017	t24	0.635	0.653	0.644	0.013	1.98%
	t0	0.660	0.664	0.662	0.003	0.43%
35.941	t24	0.607	0.662	0.635	0.039	6.13%
	t0	0.663	0.663	0.663	0.000	0.00%
57.505	t24	0.637	0.636	0.637	0.001	0.11%
	t0	0.685	0.703	0.694	0.013	1.83%
118.604	t24	0.715	0.708	0.712	0.005	0.70%
	t0	0.698	0.699	0.699	0.001	0.10%
179.703	t24	0.718	0.721	0.720	0.002	0.29%

Summary of Results

Conc.	Statistics	Time (hours)	
		0	24
0.00	Mean	0.669	0.201
	CV%	1.38%	3.52%
0.220	Mean	0.661	0.173
	CV%	0.21%	20.44%
0.661	Mean	0.667	0.145
	CV%	0.00%	8.78%
22.017	Mean	0.677	0.644
	CV%	1.88%	1.98%
35.941	Mean	0.662	0.635
	CV%	0.43%	6.13%
57.505	Mean	0.663	0.637
	CV%	0.00%	0.11%
118.604	Mean	0.694	0.712
	CV%	1.83%	0.70%
179.703	Mean	0.699	0.720
	CV%	0.10%	0.29%

Percentage Inhibition Computation

Conc.	0	100%	% I
0.00	0.201	0.468	0.000
0.220			-4.385
0.661			-11.658
22.017			92.941
35.941			94.118
57.505			94.332
118.604			103.743
179.703			104.492

Concentration vs. Percent Inhibition

Log Conc.	I%	Conc.
-0.658	-4.39	0.220
-0.180	-11.66	0.661
1.343	92.94	22.017
1.556	94.12	35.941
1.760	94.33	57.505
2.074	103.74	118.604
2.255	104.49	179.703

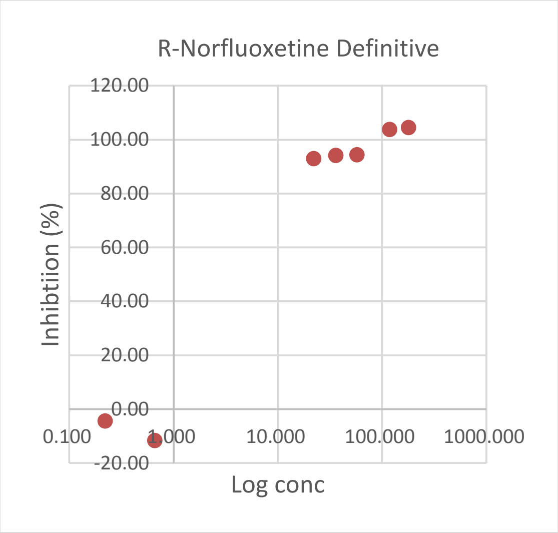

Effect Concentration Results

**log x =**	**-0.132**
**24hEC10 =**	**0.738**
**log x =**	**0.093**
**24hEC20 =**	**1.237**
**log x =**	**0.767**
**24hEC50 =**	**5.839**
**log x =**	**1.217**
**24hEC70 =**	**16.427**
**log x =**	**1.667**
**24hEC90 =**	**46.218**

Lower 95%	Upper 95%
-4.662	36.430

Summary Output

Regression Statistics	
Multiple R	0.969
R Square	0.938
Adjusted R Square	0.926
Standard Error	14.145
Observations	7

ANOVA

	df	*SS*	*MS*	*F*	*Significance F*
Regression	1	15190.518	15190.518	75.923	0.000
Residual	5	1000.391	200.078		
Total	6	16190.909			

	Coefficients	*Standard Error*	*t Stat*	*P-value*	*Lower 95%*	*Upper 95%*	*Lower 95.0%*	*Upper 95.0%*
Intercept	15.884	7.993	1.987	0.104	-4.662	36.430	-4.662	36.430
X Variable 1	44.469	5.104	8.713	0.000	31.350	57.588	31.350	57.588

Table S13. *S*-NFL Range

Results – Optical Density

Conc.	Time	Replicate 1	Replicate 2	Mean	Std. dev.	CV%
	t0	0.627	0.632	0.630	0.004	0.56%
Control	t24	0.251	0.184	0.218	0.047	21.78%
	t0	0.611	0.619	0.615	0.006	0.92%
0.005	t24	0.350	0.337	0.344	0.009	2.68%
	t0	1.123	0.610	0.867	0.363	41.86%
0.050	t24	0.794	0.177	0.486	0.436	89.86%
	t0	0.610	0.678	0.644	0.048	7.47%
0.500	t24	0.300	0.249	0.275	0.036	13.14%
	t0	0.619	0.622	0.621	0.002	0.34%
5.000	t24	0.380	0.364	0.372	0.011	3.04%
	t0	0.643	0.636	0.640	0.005	0.77%
50.000	t24	0.666	0.661	0.664	0.004	0.53%

Summary of Results

Conc.	Statistics	Time (hours) 0	24
Control	Mean	0.630	0.218
	CV%	0.56%	21.78%
0.005	Mean	0.615	0.344
	CV%	0.92%	2.68%
0.050	Mean	0.867	0.486
	CV%	41.86%	89.86%
0.500	Mean	0.644	0.275
	CV%	7.47%	13.14%
5.000	Mean	0.621	0.372
	CV%	0.34%	3.04%
50.000	Mean	0.640	0.664
	CV%	0.77%	0.53%

Percentage Inhibition Computation

Conc.	0	100%	% I
Control	0.218	0.412	0.000
0.005			34.102
0.050			7.524
0.500			10.316
5.000			39.684
50.000			105.825

Concentration vs. Percent Inhibition

Log Conc.	I%	Conc.
-2.301	34.10	0.005
-1.301	7.52	0.050
-0.301	10.32	0.500
0.699	39.68	5.000
1.699	105.83	50.000

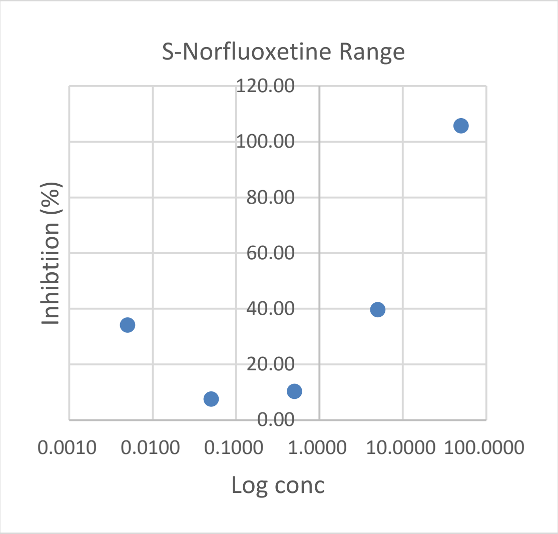

Effect Concentration Results

**log x =**	**-1.980**
**24hEC10 =**	**0.011**
**log x =**	**-1.411**
**24hEC20 =**	**0.039**
**log x =**	**0.297**
**24hEC50 =**	**1.982**
**log x =**	**1.436**
**24hEC70 =**	**27.211**
**log x =**	**2.575**
**24hEC90 =**	**373.572**

Lower 95%	Upper 95%
-2.885	92.439

Summary Output

Regression Statistics	
Multiple R	0.700
R Square	0.489
Adjusted R Square	0.319
Standard Error	32.755
Observations	5

ANOVA

	df	*SS*	*MS*	*F*	*Significance F*
Regression	1	3083.775	3083.775	2.874	0.189
Residual	3	3218.616	1072.872		
Total	4	6302.391			

	Coefficients	*Standard Error*	*t Stat*	*P-value*	*Lower 95%*	*Upper 95%*	*Lower 95.0%*	*Upper 95.0%*
Intercept	44.777	14.977	2.990	0.058	-2.885	92.439	-2.885	92.439
X Variable 1	17.561	10.358	1.695	0.189	-15.403	50.524	-15.403	50.524

Table S14. *S*-NFL Definitive

Results – Optical Density

Conc.	Time	Replicate		Mean	Std. dev.	CV%
		1	2			
	t0	0.654	0.650	0.652	0.003	0.43%
0.000	t24	0.165	0.201	0.183	0.025	13.91%
	t0	0.641	0.637	0.639	0.003	0.44%
0.093	t24	0.206	0.203	0.205	0.002	1.04%
	t0	0.646	0.654	0.650	0.006	0.87%
0.279	t24	0.225	0.254	0.240	0.021	8.56%
	t0	0.664	0.677	0.671	0.009	1.37%
9.302	t24	0.339	0.335	0.337	0.003	0.84%
	t0	0.645	0.646	0.646	0.001	0.11%
15.352	t24	0.388	0.408	0.398	0.014	3.55%
	t0	0.659	0.661	0.660	0.001	0.21%
24.562	t24	0.566	0.568	0.567	0.001	0.25%
	t0	0.682	0.674	0.678	0.006	0.83%
50.660	t24	0.680	0.686	0.683	0.004	0.62%
	t0	0.702	0.692	0.697	0.007	1.01%
76.758	t24	0.733	0.702	0.718	0.022	3.06%

Summary of Results

Conc.	Statistics	Time (hours)	
		0	24
0.00	Mean	0.652	0.183
	CV%	0.43%	13.91%
0.093	Mean	0.639	0.205
	CV%	0.44%	1.04%
0.279	Mean	0.650	0.240
	CV%	0.87%	8.56%
9.302	Mean	0.671	0.337
	CV%	1.37%	0.84%
15.352	Mean	0.646	0.398
	CV%	0.11%	3.55%
24.562	Mean	0.660	0.567
	CV%	0.21%	0.25%
50.660	Mean	0.678	0.683
	CV%	0.83%	0.62%
76.758	Mean	0.697	0.718
	CV%	1.01%	3.06%

Percentage Inhibition Computation

Conc.	0	100%	% I
0.00	0.183	0.469	0.000
0.093			7.356
0.279			12.473
9.302			28.891
15.352			47.228
24.562			80.171
50.660			101.066
76.758			104.371

Concentration vs. Percent Inhibition

Log Conc.	I%	Conc.
-1.032	7.36	0.093
-0.554	12.47	0.279
0.969	28.89	9.302
1.186	47.23	15.352
1.390	80.17	24.562
1.705	101.07	50.660
1.885	104.37	76.758

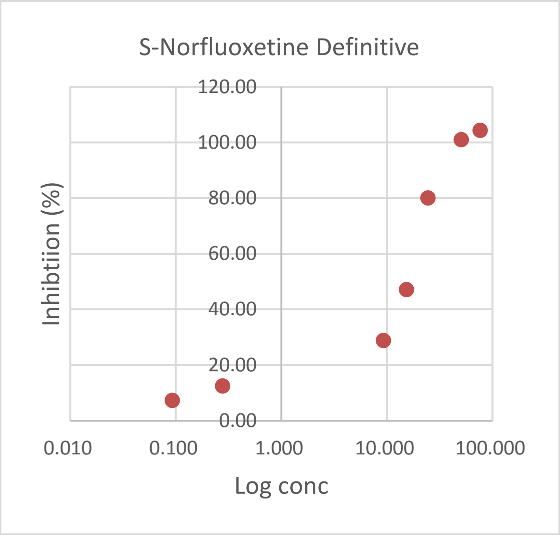

Effect Concentration Results

**log x =**	**-0.594**
**24hEC10 =**	**0.255**
**log x =**	**-0.282**
**24hEC20 =**	**0.523**
**log x =**	**0.652**
**24hEC50 =**	**4.483**
**log x =**	**1.275**
**24hEC70 =**	**18.785**
**log x =**	**1.898**
**24hEC90 =**	**78.719**

Lower 95%	Upper 95%
4.315	53.803

Summary Output

Regression Statistics	
Multiple R	0.891
R Square	0.794
Adjusted R Square	0.753
Standard Error	20.322
Observations	7

ANOVA

	df	*SS*	*MS*	*F*	*Significance F*
Regression	1	7947.019	7947.019	19.243	0.007
Residual	5	2064.954	412.991		
Total	6	10011.973			

	Coefficients	*Standard Error*	*t Stat*	*P-value*	*Lower 95%*	*Upper 95%*	*Lower 95.0%*	*Upper 95.0%*
Intercept	29.059	9.626	3.019	0.029	4.315	53.803	4.315	53.803
X Variable 1	32.104	7.319	4.387	0.007	13.291	50.918	13.291	50.918

Table S15. Structures, molecular formula, molecular weight and pK_a of selected compounds.

Compound	Chemical structure	Molecular formula	Mw (g mol^{-1})	pK_a
R-(-)-Fluoxetine (*R*-FL)	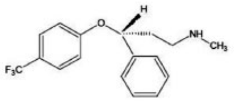	$C_{17}H_{18}F_3NO$	309.33	10.05
S-(+)-Fluoxetine (*S*-FL)	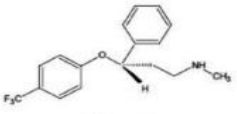			
R-(-)-Norfluoxetine (*R*-NFL)	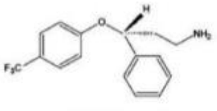	$C_{16}H_{16}F_3NO$	295.30	9.05
S-(+)-Norfluoxetine (*S*-NFL)	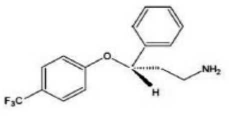			

Table S16. Physicochemical parameters (dissolved oxygen (DO), pH, temperature, total suspended solids (TSS), NO_2^-, NH_4, and chemical oxygen demand (COD)) during the river water simulating microcosms under darkabiotic (DAR), dark biotic (DBR), light abiotic (LAR) and light biotic (LBR) conditions

	D1	D2	D3	D4	D5	D8	D9	D10	D11	D12	D15	D16
pH												
DBR	8.16	8.07	7.41	7.32	7.41	7.94	8.14	8.06	7.85	7.91	8.40	8.47
DAR	8.31	8.58	8.74	8.88	8.49	8.20	8.15	7.91	8.00	7.95	8.38	8.38
LBR	8.40	8.79	7.98	7.92	7.70	8.20	8.08	8.33	8.07	8.31	8.41	8.46
LAR	8.38	8.84	8.94	8.97	8.38	8.36	8.27	8.46	8.14	8.44	8.40	8.42
DO (mg L^{-1})												
DBR	8.00	5.56	5.07	5.15	4.69	4.98	5.01	4.53	4.66	4.36	4.56	5.28
DAR	6.90	5.03	5.02	4.37	4.58	4.81	4.83	4.67	4.53	4.51	4.40	5.99
LBR	6.47	4.95	4.53	4.27	4.12	4.74	3.77	3.52	3.50	3.35	3.69	3.73
LAR	7.35	4.94	4.51	4.42	4.75	4.54	4.30	4.08	4.01	3.82	4.30	4.08
T (ºC)												
DBR	33.00	35.30	36.20	34.50	34.55	35.65	35.50	37.50	36.95	36.90	37.20	33.70
DAR	33.05	35.25	37.30	34.80	36.30	36.45	36.35	36.00	35.70	34.40	38.40	35.85
LBR	34.40	36.90	38.65	39.45	40.00	40.50	41.40	42.75	43.45	45.05	41.25	41.45
LAR	34.10	35.85	37.70	38.50	39.15	38.35	39.35	40.90	40.65	41.45	39.15	39.55
TOC (mg L^{-1})	n.a.											
TSS (g L^{-1})	0.006											
NO_2^- (mg L^{-1})	<0.02											
NH_4 (mg L^{-1})	4.3											
COD (mg L^{-1})	<25											

Table S17. Physicochemical parameters (Dissolved oxygen (DO), pH, temperature, total suspended solids (TSS), NO_2^-, NH_4, and chemical oxygen demand (COD)) during the activated sludge simulating microcosm under dark abiotic (DAR).

	0min	30min	60min	90min	2h	3h	5h	8h	12h	24h
pH										
'No spike'	7.88	8.01	7.97	8.06	7.91	8.15	7.94	8.22	8.61	7.71
10 µg L^{-1}	7.75	7.80	7.84	7.97	7.77	8.05	8.00	8.15	8.30	8.35
100 µg L^{-1}	6.96	7.82	7.98	7.98	8.02	8.18	8.27	8.21	8.42	7.37
DO (mg L^{-1})										
'No spike'	2.01	8.75	9.11	9.15	9.3	9.50	8.51	9.35	9.15	8.71
10 µg L^{-1}	1.91	8.93	9.02	9.9	9.09	8.08	8.08	8.64	8.52	8.21
100 µg L^{-1}	1.98	8.52	8.63	8.78	8.95	8.00	8.10	8.69	8.54	8.41
T (°C)										
'No spike'	16.3	15.5	17.3	18.9	20	21.6	23.0	20.8	20.8	22.6
10 µg L^{-1}	16.2	15.9	18.0	19.1	21.4	23.1	24.3	22.3	21.6	23.7
100 µg L^{-1}	16.5	15.5	17.5	19.8	21.3	23.4	24.5	22.3	21.8	24.5
TOC (mg L^{-1})										
'No spike'	2836.0	1111.53	950.18	1078.53	1629.03	1330.47	2204.43	1655.75	902.00	1464.50
10 µg L^{-1}	---	791.49	849.03	903.38	791.55	740.18	968.97	1200.75	1231.00	1823.25
100 µg L^{-1}	---	1678.78	1022.22	1734.28	1198.03	1354.47	982.47	1354.25	7742.00	1403.75
TSS (g L^{-1})										
'No spike'	2.60									
10 µg L^{-1}	2.61									
100 µg L^{-1}	2.02									
NO_2^- (mg L^{-1})	<0.02									
NH_4 (mg L^{-1})	5.2									
COD (mg L^{-1})	103									
